# Decades of dietary data demonstrate regional food web structures in the Southern Ocean

**DOI:** 10.1002/ece3.7017

**Published:** 2020-12-09

**Authors:** Stacey A. McCormack, Jessica Melbourne‐Thomas, Rowan Trebilco, Julia L. Blanchard, Ben Raymond, Andrew Constable

**Affiliations:** ^1^ Institute for Marine and Antarctic Studies University of Tasmania Hobart Tas. Australia; ^2^ CSIRO [Commonwealth Scientific and Industrial Research Organisation] Oceans and Atmosphere Hobart Tas. Australia; ^3^ Centre for Marine Socioecology University of Tasmania Hobart Tas. Australia; ^4^ Australian Antarctic Division Department of Agriculture, Water and Environment Kingston Tas. Australia; ^5^ Australian Antarctic Program Partnership University of Tasmania Hobart Tasmania Australia

**Keywords:** ecosystem management, food web structure, network analysis, Southern Ocean

## Abstract

Understanding regional‐scale food web structure in the Southern Ocean is critical to informing fisheries management and assessments of climate change impacts on Southern Ocean ecosystems and ecosystem services. Historically, a large component of Southern Ocean ecosystem research has focused on Antarctic krill, which provide a short, highly efficient food chain, linking primary producers to higher trophic levels. Over the last 15 years, the presence of alternative energy pathways has been identified and hypotheses on their relative importance in different regions raised. Using the largest circumpolar dietary database ever compiled, we tested these hypotheses using an empirical circumpolar comparison of food webs across the four major regions/sectors of the Southern Ocean (defined as south of 40°S) within the austral summer period. We used network analyses and generalizations of taxonomic food web structure to confirm that while Antarctic krill are dominant as the mid‐trophic level for the Atlantic and East Pacific food webs (including the Scotia Arc and Western Antarctic Peninsula), mesopelagic fish and other krill species are dominant contributors to predator diets in the Indian and West Pacific regions (East Antarctica and the Ross Sea). We also highlight how tracking data and habitat modeling for mobile top predators in the Southern Ocean show that these species integrate food webs over large regional scales. Our study provides a quantitative assessment, based on field observations, of the degree of regional differentiation in Southern Ocean food webs and the relative importance of alternative energy pathways between regions.

## Graphical abstract

Understanding and detecting broadscale change in Southern Ocean ecosystems will rely on understanding the processes that differentiate and maintain food web structure and function at a regional level. Using the largest circumpolar dietary database ever compiled, we tested existing hypotheses on the relative importance of mid‐trophic level organisms in providing pathways to transfer energy to higher predators across the four major oceanic sectors of the Southern Ocean. We used network analyses and generalizations of taxonomic food web structure to confirm that while Antarctic krill are dominant in the mid‐trophic level for the Atlantic and East Pacific food webs, mesopelagic fish and other krill species are dominant contributors to predator diets in the Indian and West Pacific regions.

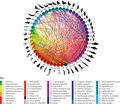

## 1 INTRODUCTION

Southern Ocean food webs are of major importance to humans and the global system, underpinning diverse values and services including the existence of wildlife populations, high‐value fisheries, and carbon sequestration (Grant et al., 2013). Historically, the emphasis of food web studies in Antarctica has related to the dominance of *Euphausia superba* (Antarctic krill) and the dependencies of so many of the Southern Ocean predators on that species (Brasier et al., 2019; El‐Sayed, 1994; Murphy et al., 2012). Since then, these food webs have become recognized to be taxonomically diverse, structurally complex, and extremely variable in space and time (Murphy et al., 2012). Notably, the structure of these food webs will have implications for how the combined effects of three main drivers of future change—climate change and ocean acidification, recovery of the great whales, and fisheries—will play out for the region's ecosystem services (Trebilco et al., 2020).

**FIGURE 1:**
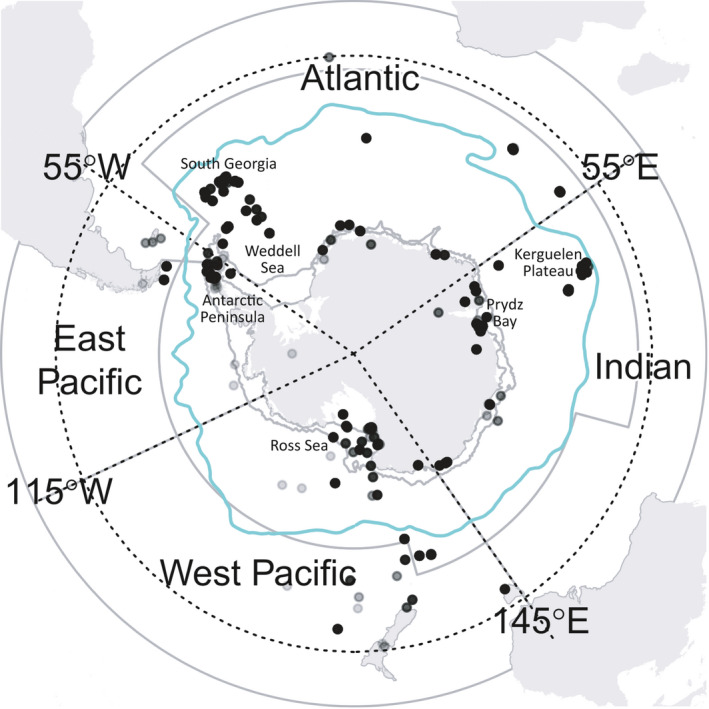
Distribution of dietary data (after refinement—see Section 2) from the SCAR Southern Ocean database across the defined Southern Ocean (south of 40°S) illustrating the boundaries of the four major oceanic sectors (following Constable et al., 2014). Transparent gray dots represent locations of diet sample data used in our analyses of food web structure with clusters represented by darker shades caused by overlayed dots. The blue line shows the polar front (Orsi et al., 1995). The gray line indicates the northern boundary of the Convention for the Conservation of Antarctic Marine Living Resources

Understanding the interactions between the three main drivers of future ecosystem change requires elaboration of plausible and justifiable food web and ecosystem models (Melbourne‐Thomas et al., 2017). The relative importance of different species in the pelagic food web with respect to the changes expected in the physical environment will vary between different sectors of the Southern Ocean (defined here as south of 40°S; see Figure 1) and will give rise to different changes in the food webs (Constable et al., 2014). An important challenge, therefore, is to determine appropriate food web structures to inform this research in different parts of the Southern Ocean (Murphy et al., 2012).

Marine ecosystem models have been, and continue to be, developed for different parts of the Southern Ocean, some of which are at much smaller spatial scales than sectors, for example, West Pacific (Ross Sea—Pinkerton et al., 2010), Indian (Prydz Bay—McCormack et al., 2019; northern Kerguelen Plateau—Subramaniam et al., 2020), Atlantic (South Georgia—Hill et al., 2012), and East Pacific (West Antarctic Peninsula—Ballerini et al., 2014; Cornejo‐Donoso & Antezana, 2008; Dahood et al., 2019; Suprenand & Ainsworth, 2017). A core component of developing these models involves representing food web interactions among species and functional groups at appropriate spatial scales.

**FIGURE 2:**
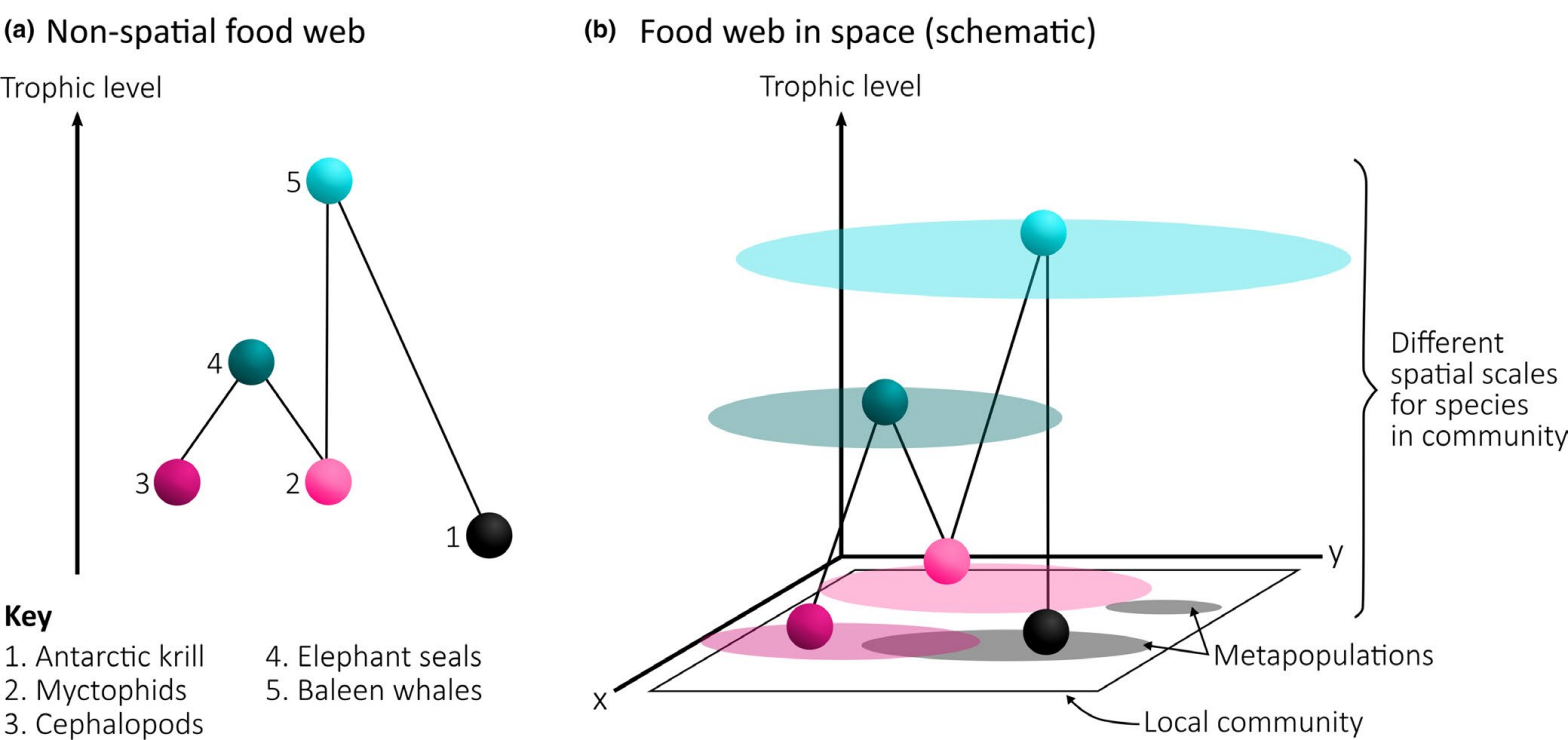
A simple food web represented (a) non‐spatially according to trophic level and (b) in space with the spatial domain relevant to the population dynamics of each species indicated by ovals. The numbers and colors of each sphere correspond to the species listed in the key. Adapted from Holt (1996)

There has been growing recognition in ecology that food webs are coupled across large scales through space and time (Albouy et al., 2019; Holt, 1996; Kortsch et al., 2018; Massol et al., 2017). Larger‐bodied predators have larger home ranges enabling them to integrate many “local” food webs or spatial patches (see, e.g., Figure 2). While regional marine ecosystem models are typically constructed at scales larger than the spatial scales at which most community ecology takes place, we propose that for the Southern Ocean, a wider macroecological lens will be informative for understanding the degree and nature of variation in food web linkages.

Construction of ecosystem models must necessarily balance the competing demands of sufficient model complexity (to adequately represent important processes) against model simplicity (e.g., to allow model parameters to be estimated from available data). Simplicity is commonly achieved by lumping species into functional groups, but this can lead to a situation in which the diet observed in an arena does not represent the population‐level diets of, and hence energy transfer to, the predators (Hill et al., 2009, 2012; Murphy et al., 2012). These issues can affect the utility of ecosystem models, and so, it is important that both the spatial scale and the taxonomic resolution of food web linkages are well‐understood to ensure ecological interactions appropriately inform model development.

Currently, there has been no quantitative assessment, based on field observations, of the degree of regional differentiation in Southern Ocean food webs or of the population‐level trophic interactions among predators and prey. Here, we use a large open‐access database consisting of 26,111 dietary observations (at the time of publication; see Section 2) to investigate variations in circumpolar food web structures, and the dominant pathways for energy flow through mid‐trophic levels for the four major oceanic sectors of the Southern Ocean. We use network analysis to explore three questions: (a) What can existing dietary observations reveal about food web structures in different sectors of the Southern Ocean? (b) What mid‐trophic level organisms provide pathways to transfer energy to higher predators in each sector of the Southern Ocean? (c) Can broad functional groups commonly used to represent Southern Ocean food webs assist in highlighting variations in food web structure between each sector? After addressing these questions, we discuss key results in the context of previous hypotheses regarding the potential structure and function of food webs in each sector and the implications for the future management of the Southern Ocean.

## 2 MATERIALS AND METHODS

For our analyses, food webs were constructed from raw diet data available from the SCAR Southern Ocean Diet and Energetics Database (SCAR, 2018). Such data are typically reported with varying degrees of taxonomic resolution, so taxa were aggregated here into appropriate groupings according to a set of logical steps (outlined below). The data were used to construct a single, overall Southern Ocean network structure, but were also separated on the basis of study location in order to construct food webs specific to each of the four major sectors of the Southern Ocean (Figure 1).

**FIGURE 3:**
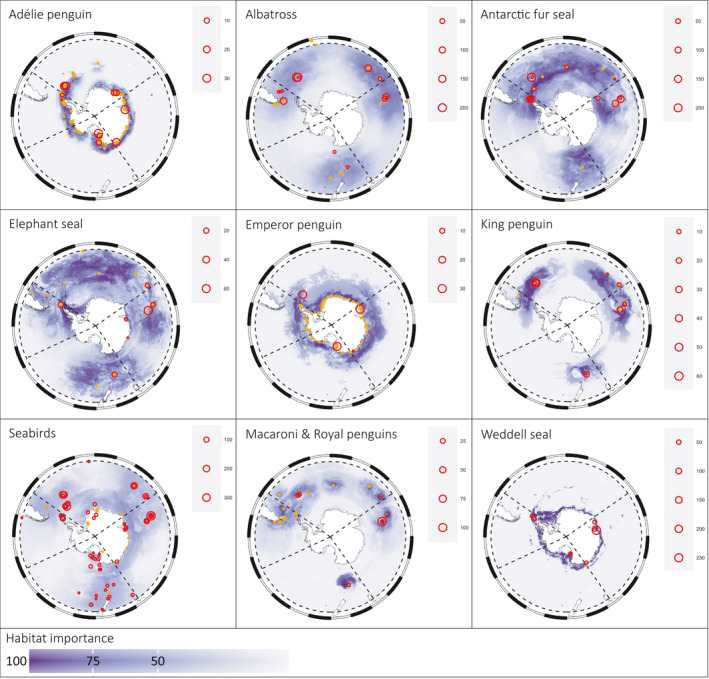
Spatial distribution of habitat importance for Southern Ocean predators, derived from Hindell et al. (2020), with Southern Ocean sectors overlaid (dashed lines). Background (purple) colors indicate habitat importance expressed in terms of area percentiles (e.g., cells with values of 90 or higher represent the top 10% most important habitat by area for that species—see Hindell et al. (2020) for details). Orange dots show the colony locations used by Hindell et al. (2020) in their habitat importance modeling. Red circles indicate the location and number of individual diet observations used in the current study. Predator groups did not exactly match the species available from Hindell et al. (2020). The albatross group includes tracking data from black‐browed, sooty, grey‐headed, light‐mantled, and wandering albatross. Seabirds includes tracking data from Antarctic and white‐chinned petrels. Note that some predators were not represented in the Hindell et al. (2020) tracking data and so do not appear in this figure

In our study, we define the Southern Ocean as the region south of 40°S, which is consistent with the delineation used in other bodies of work (e.g., De Broyer et al., 2014), although we note that there is no single, official, universally accepted definition of the area that constitutes the Southern Ocean. Recent work mapping Southern Ocean predator foraging areas (Hindell et al., 2020) indicates that the region south of 40°S encompasses areas of high habitat importance (across multiple predator species; see Figure 3), and hence defines a suitable region for the purposes of our study, in terms of representing food webs at appropriate scales. Furthermore, predator foraging habitat areas align well with the longitudinal delineations used in the definition of major Southern Ocean sectors (Figure 3, see section on “Sector‐specific food web construction” below).

### 2.1 Southern Ocean diet database

Data relating to species and their associated feeding links were obtained exclusively from the open‐access SCAR Southern Ocean Diet and Energetics Database (SCAR, 2018) (Appendix S1; Box S1). We used the dietary sample data component of the database which is a collation of 320 studies from various locations across the Southern Ocean (including records from lethal sampling of whole stomachs, stomach flushing, and scat analysis). In this study, we utilized two metrics typically used to quantify diet contributions—fraction of occurrence and fraction of diet by weight data. Fraction of occurrence is obtained through recording the number of stomachs (or scats) containing one or more items of each food category and expressing this as a percentage of the total stomachs analyzed (Hyslop, 1980). It has the advantage of being simple, and robust to variations in study methodology, but provides little indication of the relative amount of prey in each. Fraction of diet by weight is obtained from gravimetric analysis of stomach contents where the total weight of the food is determined (either wet or dry weight) and expressed as a percentage of the overall weight of the stomach contents (Hyslop, 1980). It gives a more nuanced measure of dietary importance than fraction of occurrence, but requires the prey mass be estimated from the remains present in the stomach or scat.

**FIGURE 4:**
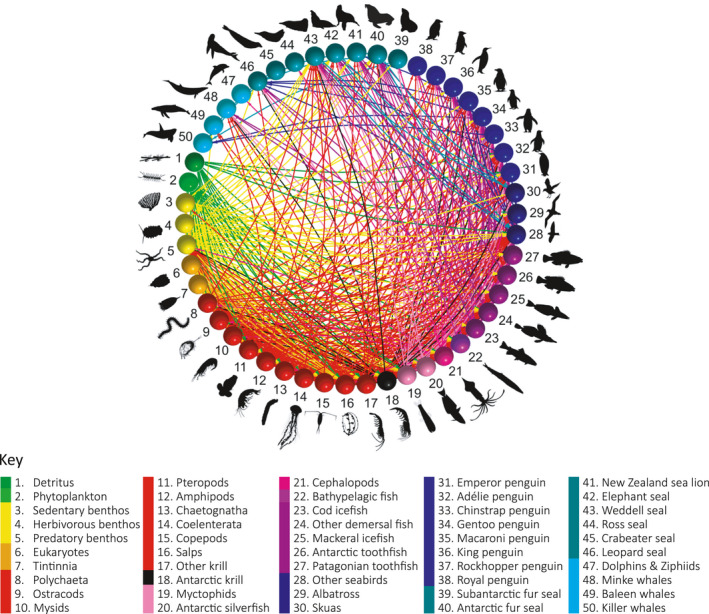
Overall network diagram generated for the 50 trophic groups and their associated interactions present in the SCAR Southern Ocean database. Nodes are colored according to broad taxonomic groups (e.g., yellow for benthic organisms, red for zooplankton) with numbers corresponding to the name of the group listed in the key. Silhouettes are representative of the types of organisms associated with each node. Edges (i.e., connections) are colored according to prey species/group and are directed toward the relevant predator node. This overall representation shows the complexity of trophic connections present in the database, which are more clearly resolved in regional food web configurations (Figure 3)

To create a Southern Ocean food web dataset, we refined the database manually by excluding data collected during the winter months (April–October), from locations north of 40°S or off the coast of the South American continent, Australia, and New Zealand where relevant (i.e., species that are not known to migrate or reside within the Southern Ocean—for example, migrating seabirds that forage within the defined Southern Ocean boundary were left in the dataset for our analysis) (Figure 1). To ensure prey species from outside the defined Southern Ocean region were not included in our analysis to the best of our ability, the distribution of each reported species in the database was checked with those with distributions outside the bounds excluded. We note that the spatial delineation of prey species is difficult within the database as many studies report prey using broad taxonomic group levels (e.g., “copepods”) which cannot be identified to specific spatial regions. Therefore, we have relied on the selection of predator species and their known foraging habitats (see, e.g., Figure 3) to further exclude observations that were likely to detail prey items from outside the bounds of the defined Southern Ocean region. We excluded nonliving entries and taxa with highly limited classifications (e.g., “Fish”) or low taxonomic resolution, as well as the associated links to those taxa (Appendix S1; Table S1). Trophic groups were constructed by aggregating taxa into groups, aiming for the finest taxonomic resolution possible given inconsistencies in data reporting and knowledge about individual species diet (Appendix S1; Table S2). The final grouping comprised 50 groups of relevance to energetic pathways through mid‐trophic levels. The groups were in some cases single species, but others comprised groups composed of similar species according to ecological characteristics (i.e., feeding and habitat—e.g., “herbivorous benthos,” “other demersal fish,” or “other seabirds”). The resulting dataset consists of 16,143 dietary observations and 410 unique predator–prey interactions among the 50 trophic groups (Figure 4).

### 2.2 Sector‐specific food web construction

The four sectors of the Southern Ocean (Figure 1) were defined following Constable et al. (2014), corresponding approximately to the four major ocean basins (Atlantic, Indian, West Pacific, and East Pacific). The boundaries of each sector were defined according to natural topographic features and biogeographical subregions of the Southern Ocean described in the literature. The Drake Passage naturally separates the Antarctic Peninsula into two regions despite being connected by the Scotia Sea. Similarly, the Macquarie Ridge separates the Indian sector from the Pacific, with the sector boundary aligned just to the west of the ridge to account for oceanographic differences to the east and west as well as northern influences of the East Australian current. The eastern extent of the influence of the Weddell Gyre and the Ross Sea Gyre, respectively, provide the boundaries between the Atlantic and Indian sectors and the West and East Pacific sectors (Constable et al., 2014; Grant et al., 2006; Kaiser et al., 2009).

**FIGURE 5:**
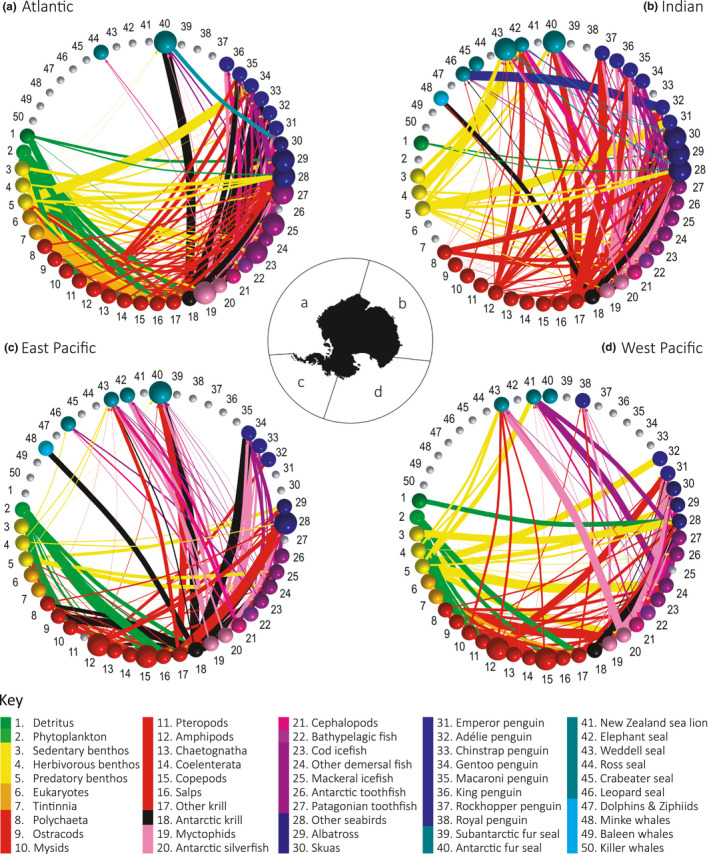
Food web network diagrams for the four major oceanic sectors of the Southern Ocean (sector boundaries represented in central Antarctic map), the (a) Atlantic sector, (b) Indian sector, (c) East Pacific sector, and (d) West Pacific sector. Colors and numbers correspond to those listed within the key. Node size is indicative of the number of species aggregated within each group and the edge width corresponds to the average fraction of occurrence of the trophic interaction between the two nodes/groups as reported in the SCAR Southern Ocean Diet and Energetics Database. Gray nodes indicate no fraction of occurrence data are currently available for the associated group in the database with other nodes colored according to broad taxonomic groups (e.g., yellow for benthic organisms, red for zooplankton). Edges (i.e., connections) are colored according to prey species/group and are directed toward the relevant predator node

We assembled cumulative food webs for each of the four major oceanic sectors of the Southern Ocean (Figure 5) based on the previously defined overall network structure (Figure 4). The food webs are cumulative over time and space as fine temporal and spatial resolution is not currently achievable. In a cumulative food web, trophic interactions are integrated across spatial and temporal scales such that the focus is on detailing energetic links among taxa that coexist within an ecosystem and have the opportunity to interact over some span of ecological time (Maschner et al., 2009). Cumulative webs are widely used for comparative purposes, in particular to investigate regularities in food web structure (Dunnes et al., 2008). There is currently very limited information on the feeding behavior of smaller organisms such as plankton and microbes in the database, and so, our food webs represent higher trophic level groups with better detail. Higher trophic level species (marine mammals and birds) were kept at species‐level where possible to provide a clearer representation of the pathways through mid‐trophic levels to associated predators. In cases where predator species were not present in every sector, we kept the data that were available in the dataset as we considered this a more realistic representation of the food web than completely excluding these groups. This methodological choice is taken into account in our interpretation and comparison of the regional food webs.

To construct food webs, we refined the Southern Ocean food web dataset to create four sector‐specific datasets that contained region‐specific diet observations (Table 1; Figure 5). We refined each dataset to exclude trophic links that had a missing fraction of occurrence value, that is, the frequency that the prey items occur in the diets of associated predators. The remaining information on trophic links was used to create weighted network diagrams using the R‐package igraph (Csardi & Nepusz, 2006) for each sector with the weight of the edges corresponding to the average fraction of occurrence value for each trophic interaction (Figure 5) (Appendix S2).

**TABLE 1:** Summary of sector‐specific food web datasets including two network structure properties, connectance (C) and average link density (LD)

Oceanic sector	Region (south of 40°S)	Data sources (number of published and unpublished studies)	Total number of Observations	Number of unique predator–prey interactions	C	LD
Atlantic	55°W–55°E	64	2,357	230	0.17	6.03
Indian	55°E–145°E	45	1,292	176	0.12	4.38
West Pacific	145°E–115°W	21	837	148	0.12	4.00
East Pacific	115°W–55°W	37	1,066	173	0.14	4.80

### 2.3 Network structure properties

A common measure used to analyze the structure of complex directed networks is the “degree” of individual nodes (Opsahl et al., 2010). An individual node in a directed network has two degrees, the in‐degree which is the total number of connections onto a node (in a food web this represents the number of other groups the node is feeding on) and the out‐degree which is the total number of connections coming from a node (i.e., the number of other groups feeding on the node) (McPherson et al., 2001; Wellman, 2008). In a weighted directed network, the strength of an individual node's degree (either in‐ or out‐degree) can be calculated by summing the weights of the associated edges (i.e., connections in or out of a node) (Newman, 2004; Opsahl et al., 2010).

**FIGURE 6:**
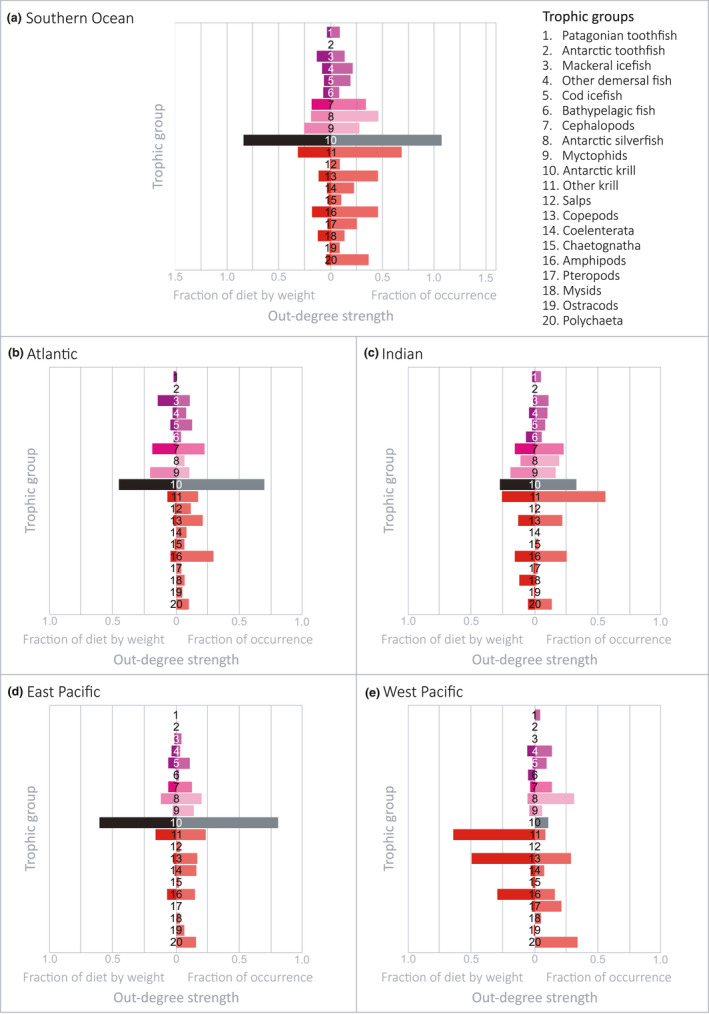
Degree strengths (out‐degree; number of trophic linkages preying upon the specific group) according to the weight of interactions (either the fraction of occurrence or fraction of diet by weight data when available) of mid‐trophic level group nodes for the (a) Southern Ocean and the food webs of the four major oceanic sectors, the (b) Atlantic sector, (c) Indian sector, (d) East Pacific sector, and (e) West Pacific sector. Trophic group numbers correspond to those listed in box (a)

To investigate the contribution of mid‐trophic level groups (from zooplankton to demersal fish) to predator diets within each sector‐specific food web, we calculated the strength of the out‐degree (i.e., the number of groups predating on the node) for each mid‐trophic level group using both the fraction of occurrence data and fraction of diet by weight data within the database to weight the associated edges (connections out of the node) (Appendix S3). The resulting strengths for each mid‐trophic group were then plotted along an axis to illustrate the variation within each sector‐specific network according to both diet metrics and across each sector of the Southern Ocean (Figure 6). This was repeated for the whole Southern Ocean food web dataset to investigate the impact of scale on interpretation (Figure 6a).

In addition, two network structure properties were calculated: the average link density (LD), which is the number of predator–prey links per trophic group; and connectance (C), which is the fraction of all possible links that are realized in the network (links/group^2^) (Dunne et al., 2002) (Table 1).

### 2.4 Simplified food web structures

**FIGURE 7:**
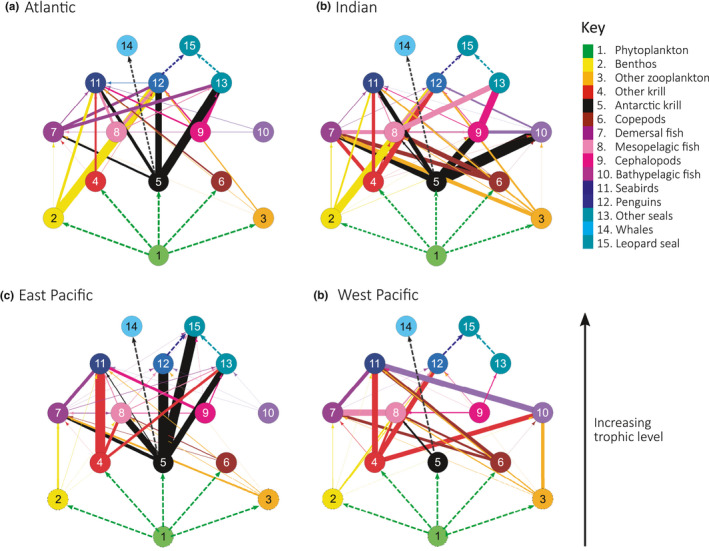
Broad food web structure for the four major oceanic sectors of the Southern Ocean, the (a) Atlantic sector, (b) Indian sector, (c) East Pacific sector, and (d) West Pacific sector. Nodes are colored and numbered according to taxonomic groups corresponding to the name of the group listed in the key. Edge widths are scaled according to fraction of diet by weight data. The color of the edges corresponds to the prey species/group and is directed toward the relevant predator node. Dashed lines indicate there is no fraction of diet by weight data associated with the interaction within the Southern Ocean Dietary Database although it is known to occur

The 50‐group networks described above provide food web representations at the finest taxonomic resolution possible given current data and ecosystem understanding. However, interpretation of these networks can be challenging, and so, we also generated further‐simplified food webs to better visualize and compare trophic connections between limited numbers of trophic levels. We aggregated the existing 50 group network structure into 15 well recognized functional groups (Figure 7), with interactions between nodes weighted according to the average of the associated fraction of diet by weight data.

### 2.5 Sampling bias

To determine whether regional differences were an artifact of sampling effort, we generated region‐specific summaries of the data used in our analyses including the number of records per individual predator species sampled (Appendix S1; Figure S1) and the fraction of total studies by each sampling methodology (Appendix S1; Figure S2). We also generated a species accumulation curve for each region‐specific dataset which is a graph of the number of species observed as a function of the sampling effort required to observe them (Colwell et al., 2004). The cumulative number of species (i.e., prey groups) were sampled randomly according to individual data sources (number of published and unpublished studies; Table 1) and plotted according to a negative exponential function (Gotelli & Colwell, 2001). The resulting curve provides an indication of the number of additional prey groups covered given additional data sources (Appendix S1; Figure S3). Each region‐specific curve gave no evidence that would suggest that regional differences in sampling effort could affect the interpretation of our results. The West Pacific sector had the lowest number of individual data sources (Table 1) although it had the fastest asymptote (Appendix S1; Figure S3), illustrating the broad group coverage by individual data sources in the West Pacific sector.

## 3 RESULTS

### 3.1 Variations in sector‐specific network properties

The total number of dietary observations (with associated fraction of occurrence data) recorded during the austral summer within the Southern Ocean diet database varied substantially between each Southern Ocean sector (Table 1). The West Pacific sector contained the lowest number of diet observations within the database (837 observations) with the Atlantic sector having the largest number of 2,357 observations. Despite differences in data availability, network structural properties were fairly consistent across sectors (Table 1). The Atlantic sector had the highest connectance value (C = 0.17) and average link density (LD = 6.03).

### 3.2 Differences in predator diets among sectors

Network pathways between groups varied between each sector‐specific food web network (Figure 5). The average fraction of occurrence associated with trophic interactions between predators and prey (represented by edge widths in Figure 5) had large variations within each sector‐specific food web.

For the Atlantic sector food web (Figure 5a), Antarctic krill had interactions with 8 out of the 11 higher predator nodes (nodes 28–50; Figure 5a), with an average fraction of occurrence greater than or equal to 50% in the diets of Adélie penguins (78%), albatross (52%), Antarctic fur seals (85%), chinstrap penguins (98%), and macaroni penguins (50%). Cephalopods had high occurrences in the diets of multiple predator groups interacting with 10 out of the 11 higher predator nodes with the highest percentage occurrence in the diets of albatross (38%), emperor penguins (66%), and king penguins (40%). Other notable interactions in the Atlantic sector food web occurred between king penguins and myctophids (37% occurrence), Adélie penguins and Antarctic silverfish (39%), and gentoo penguins and mackerel icefish (52% occurrence).

For the Indian sector food web, Antarctic krill had interactions with six out of the 15 higher predator nodes present, with an average fraction of occurrence greater than or equal to 50% in the diets of Adélie penguins (66%) and other seabirds (57%) (Figure 5b). Both amphipods and cephalopods had interactions with 12 out of the 15 higher predator nodes with all fraction of occurrence values below 50% within the two groups. The highest fraction of occurrence values were associated with other krill which interacted with 10 out of the 15 higher predator nodes with an average fraction of occurrence greater than or equal to 50% in the diets of Adélie penguins (62%), Antarctic fur seals (65%), macaroni penguins (63%), and rockhopper penguins (81%). Other notable interactions in the Indian sector food web were the occurrence of herbivorous benthos in the diet of elephant seals (92%) and Antarctic silverfish in the diets of other seabirds (85%).

In the West Pacific sector food web, Antarctic krill had interactions with three out of the nine higher predator nodes present in the network with other seabirds (71%) the only interaction with a fraction of occurrence value of more than 10% (Figure 5d). The highest fraction of occurrence values were associated with Antarctic silverfish with high occurrences in the diets of emperor penguins (95%), skuas (50%), and Weddell seals (76%). Other notable interactions occurred between emperor penguins and amphipods (66% occurrence) and New Zealand sea lions and Patagonian toothfish (42% occurrence). Cephalopods had the highest number of interactions with higher predator nodes, including six out of the nine groups, with fraction of occurrence ranging from 6% occurrence in the diet of Weddell seals to 48% in the diet of albatross species.

In the East Pacific sector food web (Figure 5c), Antarctic krill had the highest number of interactions with higher predator nodes of six out of the 10 groups present. Antarctic krill also had the highest occurrence in the diets of predators with values higher than 50% in the diets of Antarctic fur seals (84%), gentoo penguins (90%), and minke whales (52%). Other notable interactions in the East Pacific sector food web occurred between Antarctic fur seals and other krill (66% occurrence) and gentoo penguins and Antarctic silverfish (66% occurrence).

### 3.3 Pathways for energy flow through mid‐trophic levels

The out‐degree strength of mid‐trophic level group nodes varied considerably between the four sectors and Southern Ocean as a whole (Figure 6). For the Southern Ocean food web network, Antarctic krill had the largest degree strength when weighted by either fraction of occurrence data or fraction of diet by weight data. Other krill species had a significantly lower degree strength but nevertheless had the second‐largest values compared with other mid‐trophic level groups when weighted by either diet metric (Figure 6a). Other notable groups with significant degree strength were myctophids, Antarctic silverfish, and amphipods.

For the Atlantic sector network, the Antarctic krill node had the highest out‐degree strength when weighted according to either fraction of occurrence or fraction of diet by weight data (Figure 6b). The second‐highest out‐degree strength values were found for the cephalopod group node. The Indian sector network had various groups with similar out‐degree strengths. Antarctic krill had the highest out‐degree strength when weighted by fraction of diet by weight data although other krill had the highest value when weighted by fraction of occurrence data. Cephalopods, Antarctic silverfish, amphipods, and myctophids all had similar out‐degree strengths when weighted by both diet metrics (Figure 6c).

For the West Pacific network, other krill had the highest out‐degree strength when weighted by fraction of diet by weight data and Antarctic silverfish had the highest strength when weighted by fraction of occurrence data. Other high strength values when weighted by fraction of occurrence were recorded for copepods and polychaetes (Figure 6e). For the East Pacific network, the Antarctic krill node had significantly larger out‐degree strength values when weighted by both diet metrics compared with other mid‐trophic level nodes (Figure 6d).

### 3.4 Broad functional groups reveal variations in food web structure

Interactions within the simplified network structures for each sector‐specific food web (Figure 7) varied across the four sectors. The average fraction of diet by weight values associated with trophic interactions between predator and prey groups (represented by the widths of edges in Figure 7) varied among mid‐trophic level groups within each sector‐specific food web.

For the Atlantic sector (Figure 7a), Antarctic krill had direct interactions with each of the three broad predator groups (penguins, seals, and seabirds) with reasonably high fraction of diet by weight values of 60% in penguin diets, 28% in seabird diets, and 75% in seal diets on average.

In the Indian sector, Antarctic krill had both direct and indirect connections to the three predator groups (Figure 7b). Antarctic krill was directly connected to seabirds (39%) and had indirect connections to seals and penguins, connected via the intermediate groups of cephalopods (70%), mesopelagic fish (12%), and bathypelagic fish (96%). For the seal group, the most significant interactions were with cephalopods (65%) and mesopelagic fish (50%) while other krill had strong interactions with penguins (50%) and seabirds (29%).

Antarctic krill had few interactions within the West Pacific food web (Figure 7d) with the only fraction of diet by weight value being in the diet of mesopelagic fish (17%). Seabirds had large interactions with four groups: demersal fish (33%), other krill (52%), copepods (35%), and bathypelagic fish (50%). The largest fraction of diet by weight value in penguin diets was associated with other krill (44%) with little prey data available for seals with cephalopods being the only group with data available (9%).

For the East Pacific sector food web (Figure 7c), Antarctic krill had high fraction of diet by weight values associated with multiple other groups including penguins (91%), seals (56%), leopard seals (83%), and mesopelagic fish (56%). For seabirds, other krill had the largest fraction of diet by weight value in their diet (85%) with cephalopods (25%) and demersal fish (28%) also occurring in their diet.

## 4 DISCUSSION

The degree of regional differentiation in Southern Ocean food webs has remained an important but largely unresolved question for decades (Knox, 1984; Moloney & Ryan, 1995; Murphy et al., 2012). Here, we use circumpolar network analyses to provide data‐driven insights into variations in food web structure across the four major oceanic sectors of the Southern Ocean. Through analysis of network structure using available dietary metrics at various levels of complexity, we confirm that trophic groups other than Antarctic krill are the major contributors to energy flow pathways in the Indian and West Pacific sectors, consistent with previous studies (McCormack et al., 2019; Nicol & Raymond, 2012; Pinkerton et al., 2010).

In regions surrounding the Antarctic Peninsula (Atlantic and East Pacific sectors), we found that Antarctic krill dominates energy flow pathways through mid‐trophic levels (Figure 5a,c and Figure 6b,d). This is consistent with previous analyses that have identified Antarctic krill as playing the central role in the food web as the main food source (by biomass) for the majority of the higher level predators within the region (Barrera‐Oro, 2002; Clarke et al., 2007). While our findings indicate that Antarctic krill dominate food web connections between primary producers and higher trophic levels within the region for the majority of higher level predators, energy‐rich mesopelagic *Myctophidae* fish and the pelagic Antarctic silverfish (*Pleuragramma antarctica*) were important contributors to the diets of emperor, king and Adélie penguins (Figure 5a,c). This finding is consistent with previous studies that have identified these species as the second most important element of food webs surrounding the Antarctic Peninsula (Barrera‐Oro, 2002; Saunders et al., 2019). These alternative food sources might be essential in maintaining predator populations inhabiting the Antarctic Peninsula region under future environmental change. Although currently dominant, Antarctic krill is a species targeted by fisheries and potentially vulnerable to climate change impacts (Flores et al., 2012; Kawaguchi et al., 2013). As temperatures surrounding the Antarctic Peninsula continue to warm at rates faster than anywhere else on Earth (Bromwich et al., 2013), understanding the capacity of predators reliant on Antarctic krill to adapt to potential variations in food availability will be essential in predicting large scale alterations to food web structure in the region.

The available data indicate alternative network configurations in the Indian and West Pacific sectors of the Southern Ocean, where a variety of mid‐trophic level organisms, other than Antarctic krill, dominate food web connections (Figure 6c,e). Network analyses of dietary data collected from the Indian sector revealed that other krill species (other members of the family *Euphausiidae*) exceed Antarctic krill in the number of trophic interactions with predator groups during the austral summer (Figure 5b). The average strength of these interactions (Figure 6c) when weighted according to available diet metrics suggest that other krill species collectively have an equal or greater role in the diets of predator species within the Indian sector. Antarctic krill densities are generally lower in the East Antarctic region compared with the South Atlantic (Jarvis et al., 2010; Kawaguchi et al., 2010) with notable absences from inshore of the shelf break (Nicol & Raymond, 2012) and off the islands of the Kerguelen Plateau. This distinguishes East Antarctica from the South Atlantic where island groups (South Georgia, Bouvet, and South Sandwich Islands and South Orkneys) generally support Antarctic krill‐based pelagic ecosystems (Constable et al., 2000).

Unlike the Atlantic, few food web descriptions exist for East Antarctica although many studies have begun hinting at species that might be key contributors to energy flow within the Indian sector food web (McCormack et al., 2019). The shelf community off East Antarctica is generally dominated by the neritic *Euphausia crystallorophias* (Nicol & Raymond, 2012), which has previously been identified as an important prey species in the diets of breeding Adélie penguins (Puddicombe & Johnstone, 1988), crabeater seals (Hempel, 1985), and various whales. Adélie penguins in East Antarctica in particular are believed to rely on *E. crystallorophias* during their breeding season (Thomas & Green, 1988) which varies from colonies in the Antarctic Peninsula where *E. superba* dominates the diet of Adélie penguins (Nagy & Obst, 1992) (Figure 5a).

The dominant role of Antarctic krill in the West Pacific sector of the Southern Ocean has been questioned previously (Pinkerton et al., 2010) with emphasis placed on the potential role of Antarctic silverfish (*P. antarctica*), *E. crystallorophias*, and toothfish (*Dissostichus* sp.). The West Pacific sector has several unique geographical and biological features distinguishing the region from the rest of the Southern Ocean. Compared with other Antarctic regions, the Ross Sea has a wide and deep continental shelf, with the shelf break occurring at 700 m. Covered by sea ice for at least 9 months of the year, the continental shelf waters are dominated by *E. crystallorophias* and Antarctic silverfish. Antarctic krill generally only occur in the region spanning from the shelf break to the Polar Front (located at about 60°S) in the West Pacific sector. In the continental shelf region, Antarctic krill are reported to be absent (Marr, 1962) with the majority of predator species preying primarily on fish species and *E. crystallorophias* (La Mesa et al., 2004). One of the most interesting features of the West Pacific sector is a sharp decrease in temperature of the deep water (from +0.5 to −1.8°C) in the northern area of the Ross Sea resulting in an absence of almost all mesopelagic myctophids, gonostomatids, bathylagids, and paralepidids from the continental shelf (DeWitt, 1970).

Our network analyses for the West Pacific sector identified Antarctic silverfish as having the largest occurrence in diets of predator species in the region (Figures 5d, 6e). Due to the unique geographical features of the West Pacific sector, the Ross Sea food web has long been characterized as distinct from other regions of the Southern Ocean. The key role of Notothenioid fish, as both predators and as prey to majority of the higher level predators living and foraging on the shelf, generates a network of complex predator–prey interactions. Within the West Pacific food web network, Antarctic silverfish and other krill species (especially *E. crystallorophias*) are thought to have an ecological role equivalent to that of myctophids and Antarctic krill elsewhere in the Southern Ocean (La Mesa et al., 2004).

Like in all marine ecosystems, food webs in the Southern Ocean show considerable spatial (local, regional, and circumpolar) and temporal (seasonal, interannual, decadal, and longer‐term) variability in physical and biological structure and function. There are major gaps in our understanding of the seasonality of Southern Ocean food webs with difficulties associated with winter sampling resulting in few observations available outside of the summer ice‐free period. This study outlines the first circumpolar comparison of food web structure across regions of the Southern Ocean in the austral summer and provides novel methods for standardization and regional comparisons. While we recognize that the methods described are limited by the available data, we found few discrepancies between the regions that would suggest our findings are a result of sampling effort. By stratifying the Southern Ocean dietary database to isolate the most reliable estimates of diet composition, we have provided further insights into the potential structure of food webs within the austral summer in regions that previously had few to no syntheses available including the Indian and West Pacific sectors. It is important to highlight that using diet composition alone does not allow us to make inferences regarding the amount of energy that is transferred via these pathways. While we have identified important routes for energy flow within each sector, further parameters are required to determine the rate that energy can flow through each trophic pathway.

Understanding the structure and function of food webs during the winter remains one of the largest gaps in Southern Ocean ecosystems research. Increasing amounts of data are becoming available on the winter activity and diet of some predator species (Cherel et al., 1996) with new techniques such as stable isotopes, fatty acids, and DNA also beginning to provide further insight into predator–prey dynamics in the Southern Ocean (Cherel et al., 2018). Such techniques help fill gaps that result from the limitations of stomach content data. For example, gelatinous species, often underrepresented in stomach content data due to fast digestion rates, might be an essential component of Southern Ocean food webs (McInnes et al., 2017). Compiling the information gained from new techniques along with historical data yet to be incorporated into datasets such as the SCAR Southern Ocean Diet and Energetics Database remains an important goal for Southern Ocean research.

In recent decades, conceptual frameworks have emerged that recognize that no single model structure is likely to be capable of adequately capturing all aspects of Southern Ocean ecosystem dynamics (Murphy et al., 2007, 2012, 2016). Incorporating these frameworks for exploring alternative energy pathways in Southern Ocean ecosystems into future food web modeling efforts will assist in generating alternative views of the potential response of ecosystems to perturbations and allow for comparative analyses of structure and function. For example, a mass‐balance model developed for South Georgia was used to explore the potential consequences of a shift from an Antarctic krill to a copepod‐dominated system under scenarios of a warming climate (Hill et al., 2012).

Building these alternative food web models for the Southern Ocean will require more extensive data collection which raises the question of optimal sampling and monitoring strategies to understand and characterize alternative energy pathways. Our study does not consider latitudinal and seasonal variations in food web structure due to the constraints of current data availability. An important direction for future work will be to determine sampling approaches that can enable food web characterization at finer scales (such as that provided by Saunders et al., 2019) and also to consider approaches for (a) better distinguishing Antarctic from subantarctic marine food webs and (b) characterizing linkages between them. Recognizing the complexity of Southern Ocean food webs and the role of alternative energy pathways, which might vary in dominance across regions and seasons, will be essential for determining ecosystem responses to varying environmental conditions (Murphy et al., 2016).

As we move into an era where the need to guide management of marine ecosystems and resources outweighs the capacity of science to completely understand the causes and consequence of long‐term change, targeted sampling and large international cross‐disciplinary collaborative efforts will be essential (Newman et al., 2019). Developing a strong foundation for understanding the capacity of Southern Ocean ecosystems to adapt to environmental perturbations and the ever‐growing presence of humans and harvesting will require articulating circumpolar sampling designs and determining priorities for research efforts (Constable et al., 2016). Filling in “missing links” in existing food web representations and ensuring the flexibility of food web models to predict potential shifts in ecosystem state from Antarctic krill dominated systems to alternative configurations where other mid‐trophic level organisms play dominant roles (and vice versa) will be a key priority for ensuring that models can provide the information required to guide ecosystem management in a changing Southern Ocean.

## CONFLICT OF INTEREST

The authors declare that there are no conflicts of interest.

## AUTHOR CONTRIBUTIONS

**Stacey A. McCormack:** Conceptualization (lead); formal analysis (lead); visualization (lead); writing – original draft (lead); writing – review and editing (equal). **Jessica Melbourne‐Thomas:** Conceptualization (supporting); formal analysis (supporting); writing – original draft (supporting); writing – review and editing (equal). **Rowan Trebilco:** Conceptualization (supporting); formal analysis (supporting); writing – original draft (supporting); writing – review and editing (equal). **Julia L. Blanchard:** Conceptualization (supporting); writing – original draft (supporting); writing – review and editing (equal). **Ben Raymond:** Formal analysis (supporting); software (lead); visualization (supporting); writing – review and editing (supporting). **Andrew Constable:** Conceptualization (supporting); formal analysis (supporting); writing – original draft (supporting); writing – review and editing (equal).

## Supporting information

Appendix S1

Appendix S2

Appendix S3

## Data Availability

Dietary data: SCAR Southern Ocean Diet and Energetics Database https://doi.org/10.26179/5b6cd40bb6935
