# Supplementary material for: Decades of dietary data demonstrate regional food web structures in the Southern Ocean
Source: Ecol Evol. 2020 Dec 9;11(1):227–41. doi: 10.1002/ece3.7017 (PMC7790630; doi:10.1002/ece3.7017)
Supplement: Supplementary file 1 — Appendix S1 [file ECE3-11-227-s001.docx]

**Appendix S1**

**Box S1**: Overview of how to access the data available through the SCAR Southern Ocean Diet and Energetics Database.

**Accessing the SCAR Southern Ocean Diet and Energetics Database**

The R package ‘sohungry’ provides access the SCAR Southern Ocean Diet and Energetics Database and tools for working with the data. See GitHub <https://github.com/SCAR/sohungry>

**Installing**

install.packages("devtools")

library(devtools)

install_github("SCAR/sohungry")

**Usage**

In this study, the diet dataset was used. To import this data use the following code:

# Load SODD dietary data

# Loading diet data (stomach content analyses and similar) and assign variable x

x <- so_diet() # 26511 observations

**Table S1:** Dietary entries from the Southern Ocean Dietary Database classified as non-living or having highly limited information and excluded from food web analyses

| Entry name | Basis for removal | Entries removed |
| --- | --- | --- |
| *Unidentified* | No information | 528 |
| *All / all* | No information | 823 |
| *None* | No information | 112 |
| *Stones* | Non-living | 174 |
| *Egg* | Irrelevant to investigation | 18 |
| *Sediment* | Non-living | 9 |
| *Invertebrate eggs* | Irrelevant to investigation | 29 |
| *Synthetic material* | Non-living | 1 |
| *Anthropogenic material* | Non-living | 1 |
| *Organic matter* | Irrelevant to investigation | 1 |
| *Chilli peppers* | Irrelevant to investigation | 1 |
| *Longline fishing hook* | Non-living | 1 |
| *Seaweed* | Insignificant number of observations | 3 |
| *Sand* | Non-living | 1 |
| *Hair* | Irrelevant to investigation | 20 |
| *Plastic* | Non-living | 10 |
| *Gravel* | Non-living | 16 |
| *Feathers* | Irrelevant to investigation | 20 |
| *Station refuse* | Non-living | 32 |
| *Mucus* | Irrelevant to investigation | 5 |
| *Residue* | Non-living | 383 |
| *Fish eggs* | Limited information | 131 |
| *Fish scales* | Irrelevant to investigation | 35 |
| *Bait* | Non-living | 2 |
| *Hooks* | Non-living | 2 |
| *Skate egg case* | Irrelevant to investigations | 2 |
| *Filter feeding* | Non-living | 1 |
| *Paint debris* | Non-living | 1 |
| *Netting debris* | Non-living | 1 |
| *Vegetable matter* | Non-living | 3 |
| *Fecal Pellet* | Non-living | 1 |
| *Fish* | Highly aggregated / incomplete trophic information | 1656 |
| *Spheniscidae* | Highly aggregated / incomplete trophic information | 72 |
| *Phocidae* | Highly aggregated / incomplete trophic information | 6 |
| *Aves* | Highly aggregated / incomplete trophic information | 21 |
| *Invertebrata* | Highly aggregated / incomplete trophic information | 13 |
| *Animalia* | Highly aggregated / incomplete trophic information | 9 |
| *Eukaryota* | Highly aggregated / incomplete trophic information | 9 |
| *Malacostraca* | Highly aggregated / incomplete trophic information | 1 |
| *Plant material* | Highly aggregated / incomplete trophic information  All entries recorded in the stomach of cod icefish | 228 |
| *Actinopterygii* | Highly aggregated / incomplete trophic information | 21 |
| *Algae* | Incomplete trophic information | 42 |
| *Microphytobenthos* | Insignificant number of observations (< 4) | 2 |
| *Macroalgae* | Insignificant number of observations (< 4) | 8 |
| *Otariidae* | Highly aggregated / incomplete trophic information | 3 |
| *Insecta* | Highly aggregated / incomplete trophic information | 11 |
| *Teleostei* | Highly aggregated / incomplete trophic information | 7 |
| *Vertebrata* | Highly aggregated / incomplete trophic information | 5 |
| *Halacaroidea* | Highly aggregated / incomplete trophic information | 1 |
| *Annelida* | Highly aggregated / incomplete trophic information | 10 |
| *Nemertea* | Highly aggregated / incomplete trophic information | 4 |
| *Sipuncula* | Highly aggregated / incomplete trophic information | 4 |
| *Nabis capsiformis* | Terrestrial insect - Not found in Southern Ocean | 2 |
| *Pyraloidea* | Terrestrial insect - Not found in Southern Ocean | 1 |
| *Noctuidae* | Terrestrial insect - Not found in Southern Ocean | 1 |
| *Santia bicornis* | Terrestrial insect - Not found in Southern Ocean | 7 |
| *Lanceola sp.* | Not found in Southern Ocean | 1 |
| *Aega semicarinata* | Not found in Southern Ocean | 1 |
| *Mammalia* | Highly aggregated / incomplete trophic information | 9 |
| *Parasitic worms* | Parasites removed for our investigations | 8 |
| *Eumetazoa* | Highly aggregated / incomplete trophic information | 17 |
| *Dendrobranchiata* | Highly aggregated / incomplete trophic information | 2 |
| *Parasites* | Parasites removed for our investigations | 3 |
| *Laminariales* | Insignificant number of observations (< 4) | 3 |
| *Acanthocephala* | Parasites removed for our investigations | 18 |
| *Cestoda* | Parasites removed for our investigations | 1 |
| *Cladocera* | Freshwater species | 4 |
| *Larvacea* | Highly aggregated / incomplete trophic information | 27 |
| *Micromesistius australis* | Insignificant number of observations (≤4) | 4 |
| *Sardinops sagax* | Insignificant number of observations (≤4) | 1 |
| *Scomberesox saurus* | Not found in the Southern Ocean | 32 |
| *Lamna nasus* | Not typically found in the Southern Ocean | 15 |
| *Procellariiformes* | Highly aggregated / incomplete trophic information | 8 |
|  | **Total Entries Removed** | **4750** |

**Table S2**: Taxonomic group classification, definition and included species used in the 50 trophic-group Southern Ocean food web dataset

| Taxonomic group | Group definition | Species / groups included (as they appear in the database) | Common Names | Resolution |
| --- | --- | --- | --- | --- |
| Detritus | Waste or debris, includes water-column and sediment detritus | *Detritus*  *Euphausiacea moult debris* |  |  |
| Carrion | Dead animal matter | *Carrion*  *Offal* |  |  |
| Phytoplankton | Autotrophic plankton species | *Phytoplankton*  *Phytoplankton and detritus*  *Coscinodiscophycidae*  *Bacillariophyta*  *Pennales*  *Coscinodiscophyceae*  *Fragilariopsis sp.*  *Eucampia sp.*  *Nitzschia sp.*  *Amphiprora kufferathii*  *Berkeleya rutilans*  *Navicula directa*  *Navicula glaciei*  *Nitzschia obliquecostata*  *Amphora sp.*  *Gomphonema sp.*  *Navicula sp.*  *Synedra sp.*  *Porosira pseudodenticulata*  *Thalassiosira australis*  *Thalassiosira sp.*  *Fragilariopsis curta*  *Pinnularia quadratarea*  *Pleurosigma directum*  *Fragilariopsis cylindrus*  *Chaetoceros sp.*  *Eucampia antarctica*  *Corethron criophilum*  *Corethron sp.*  *Dinophyceae*  *Prorocentrum sp.*  *Protoperidinium sp.*  *Dinophysis sp.*  *Peridiniales*  *Distephanus speculum*  *Dictyochophyceae*  *Dictyochales*  *Silicoflagellates* | Phytoplankton  Phytoplankton  Diatoms  Diatoms  Diatoms  Diatoms  Diatoms  Diatoms  Diatoms  Diatoms  Diatoms  Diatoms  Diatoms  Diatoms  Diatoms  Diatoms  Diatoms  Diatoms  Diatoms  Diatoms  Diatoms  Diatoms  Diatoms  Diatoms  Diatoms  Diatoms  Diatoms  Diatoms  Diatoms  Dinoflagellates  Dinoflagellates  Dinoflagellates  Dinoflagellates  Dinoflagellates  Silicoflagellates  Silicoflagellates  Silicoflagellates  Silicoflagellates | Subclass  Class  Order  Class  Genus  Genus  Genus  Species  Species  Species  Species  Species  Genus  Genus  Genus  Genus  Species  Species  Genus  Species  Species  Species  Species  Genus  Species  Species  Genus  Class  Genus  Genus  Genus  Order  Species  Class  Order  Class |
| Sedentary benthos | Members of the following Phylum:   - Porifera - Bryozoa - Cnidaria - Chordata | *Cymodocella sp.*  *Porifera*  *Tetilla leptoderma*  *Rossella racovitzae*  *Haliclona dancoi*  *Homaxinella balfourensis*  *Mycale acerata*  *Haliclona penicillata*  *Polymastia invaginata*  *Haliclona (Gellius) tenella*  *Calyx arcuarius*  *Isodictya setifera*  *Kirkpatrickia variolosa*  *Haliclona pedunculata*  *Bryozoa*  *Cibacapsa gulosa*  *Coral*  *Scleractinia*  *Clavularia frankliniana*  *Actiniaria*  *Tunicata*  *Ascidiacea*  *Clavularia cf. frankliniana*  *Anthomastus bathyproctus*  *Urticinopsis antarctica* | Seagrass  Sponges  NA  NA  NA  NA  NA  NA  NA  NA  NA  NA  NA  NA  Moss animals  NA  Coral  Stony corals  NA  Sea anemones  NA  NA  NA  NA  Sea anemone | Genus  Phylum  Species  Species  Species  Species  Species  Species  Species  Species  Species  Species  Species  Species  Phylum  Species  Class  Order  Species  Order  Subphylum  Class  Species  Species  Species |
| Herbivorous benthos | Members of the following Phylum:   - Mollusca - Nermertea - Nematoda - Brachiopoda - Sipuncula   & Benthic species of the order Isopoda | *Gastropoda*  *Eatoniella sp.*  *Nacella concinna*  *Rissoa sp.*  *Naticidae*  *Trophon sp.*  *Opisthobranchia*  *Nacella macquariensis*  *Laevilitorina caliginosa*  *Kerguelenella lateralis*  *Nudibranchia*  *Photinula expansa*  *Philine alata*  *Toledonia sp.*  *Armodoris antarctica*  *Powellisetis deserta*  *Iothia coppingeri*  *Cephalaspidea*  *Buccinidae*  *Cantharidus sp.*  *Nemertea*  *Lepas sp.*  *Lepas australis*  *Lepas (Anatifa) australis*  *Nauticaris sp.*  *Balanomorpha*  *Cirripedia*  *Laternula elliptica*  *Bivalvia*  *Genaxinus bongraini*  *Adamussium colbecki*  *Hemiarthrum setulosum*  *Lasaea consanguinea*  *Gaimardia trapesina*  *Kidderia minuta*  *Lepas australis*  *Laternula elliptica*  *Adamussium colbecki*  *Limatula hodgsoni*  *Mollusca*  *Polyplacophora*  *Placophora*  *Nematoda*  *Brachiopoda*  *Sipuncula*  *Isopoda*  *Anuropus sp.*  *Glyptonotus antarcticus*  *Valvifera*  *Natatolana sp.*  *Glyptonotus sp.*  *Dynamenella huttoni*  *Exosphaeroma gigas*  *Gnathiidae*  *Sphaeromatidae*  *Bopyrid*  *Arcturides cornutus*  *Antarcturus sp.*  *Septemserolis septemcarinata*  *Serolidae*  *Spinoserolis latifrons*  *Cassidinopsis emarginata*  *Serolis sp.*  *Eurycope sp.*  *Aega antarctica*  *Munna antarctica*  *Gnathia sp.*  *Munnidae*  *Arcturidae*  *Anuropus antarcticus*  *Tanaidacea*  *Cumacea*  *Leptostraca*  *Neognathophausia gigas*  *Neognathophausia ingens*  *Gnathophausia*  *Pseudochalaraspidum sp.*  *Leptognathia sp.*  *Doris kerguelenensis*  *Tritoniella belli*  *Notaeolidia gigas*  *Aeolidida*  *Doto sp.*  *Trophon longstaffi* | Snails & Slugs  NA  Sea snails  NA  Moon snails  Sea snails  NA  Sea snails  Sea snails  Limpet  Nudibranchs  Sea snails  NA  Sea snails  Sea slugs  NA  Sea snails  Headshield slugs  Whelks  NA  Ribbon worms  Goose barnacles  Goose barnacles  Goose barnacles  NA  Barnacles  Barnacles  Saltwater clam  NA  NA  Bivalve  Chiton  Bivalve  NA  NA  NA  NA  NA  NA  NA  Chitons  Chitons  Roundworms  NA  Unsegmented worms  NA  NA  NA  NA  NA  NA  NA  NA  NA  NA  NA  NA  NA  NA  NA  NA  NA  NA  NA  NA  NA  NA  NA  NA  NA  NA  Hooded shrimp  NA  NA  NA  NA  NA  NA  Sea slug  Sea slug  Sea slug  Sea slugs  NA  NA | Class  Genus  Species  Genus  Family  Genus  Infraclass  Species  Species  Species  Family  Species  Species  Genus  Species  Species  Species  Order  Family  Species  Phylum  Genus  Species  Species  Genus  Suborder  Infraclass  Species  Phylum  Species  Species  Species  Species  Species  Species  Species  Species  Species  Species  Phylum  Class  Class  Phylum  Phylum  Phylum  Order  Genus  Species  Suborder  Genus  Genus  Species  Species  Family  Family  Family  Species  Genus  Species  Family  Species  Species  Genus  Genus  Species  Species  Genus  Family  Family  Species  Order  Order  Order  Species  Species  Genus  Genus  Genus  Species  Species  Species  Infraorder  Genus  Species |
| Predatory benthos | Members of the following groups:   - Echinodermata - Crustacea - Priapulida - Decapoda | *Echinodermata*  *Labidiaster annulatus*  *Ophionotus hexactis*  *Ophionotus victoriae*  *Ophiuroidea*  *Sterechinus neumayeri*  *Echinoidea*  *Promachocrinus kerguelensis*  *Crinoidea*  *Asteroidea*  *Cidaroida*  *Holothuroidea*  *Anasterias rupicola*  *Odontaster validus*  *Bathyplotes sp.*  *Echinocucumis sp.*  *Crustacea*  *Pterygosquilla armata*  *Pycnogonida*  *Pantopoda*  *Pentanymphon antarcticum*  *Endeis australis*  *Austrodecus sp.*  *Endeidae*  *Priapulida*  *Decapoda*  *Pasiphaea sp.*  *Pasiphaea scotiae*  *Notocrangon antarcticus*  *Notocrangon*  *Chorismus antarcticus*  *Gennadas sp.*  *Nematocarcinus sp.*  *Halicarcinus planatus*  *Austropandalus grayi*  *Parapasiphae sp.*  *Sergestes sp.*  *Acanthephyra sp.*  *Pasiphaeidae*  *Caridea*  *Lithodidae*  *Nectocarcinus antarcticus*  *Acanthephyra pelagica*  *Acanthephyra sp.*  *Pagurus sp.*  *Brachyura*  *Ophiosparte gigas*  *Odontaster meridionalis*  *Acodontaster conspicuus*  *Acodontaster hodgsoni*  *Perknaster fuscus antarcticus*  *Diplasterias brucei*  *Macroptychaster accrescens*  *Ammothea striata*  *Colossendeis sp.*  *Nauticaris marionis*  *Echiuroidea* | NA  Starfish  Starfish  Starfish  Brittle star  Antarctic sea urchin  Sea urchin  NA  Crinoid  Starfish  Sea urchin  Sea cucumber  Starfish  Starfish  NA  NA  NA  NA  Sea spider  Sea spiders  NA  NA  NA  NA  Penis worms  NA  Pasiphaeid shrimp  NA  NA  NA  NA  Penaeid shrimp  NA  Flattened crab  NA  NA  NA  NA  NA  Caridean shrimp  King crabs  NA  NA  NA  Hermit crabs  Crab  NA  Starfish  Starfish Starfish  Starfish  Starfish  Starfish  Sea spider  Sea spider  NA  Spoon worms | Phylum  Species  Species  Species  Class  Species  Class  Species  Class  Class  Order  Class  Species  Species  Genus  Genus  Subphylum  Species  Class  Order  Species  Species  Genus  Family  Phylum  Order  Genus  Species  Species  Genus  Species  Genus  Genus  Species  Species  Genus  Genus  Genus  Family  Infraorder  Family  Species  Species  Genus  Genus  Infraorder  Species  Species  Species  Species  Species  Species  Species  Species  Genus  Species  Order |
| Eukaryotes | Organisms classified as Eukaryote | *Protozoa*  *Radiolaria*  *Heliozoa*  *Foraminifera*  *Sticholonche sp.* | NA  NA  Sun-animalcules  Amoeboid protists  Radiolarians | Kingdom  Subphylum  Order  Phylum  Genus |
| Tintinnia | Members of the order Tintinnidae | *Tintinnidae*  *Codonellopsis sp.*  *Cymatocylis sp.*  *Codonellopsis gaussi*  *Laackmanniella naviculaefera*  *Cymatocylis antarctica* | Tintinnia  NA  NA  NA  NA  NA | Order  Genus  Genus  Species  Species  Species |
| Polychaeta | Members of the class Polychaeta | *Polychaeta*  *Pelagobia sp.*  *Aglaophamus sp.*  *Ampharetidae*  *Eunice sp.*  *Nephtyidae*  *Onuphidae*  *Phyllodoce sp.*  *Phyllodocidae*  *Polynoidae*  *Rhamphobrachium sp.*  *Trichobranchidae*  *Lumbrineridae*  *Maldanidae*  *Spionidae*  *Cirratulidae*  *Pelagobia longicirrata*  *Alciopidae*  *Tomopteris sp.*  *Rhynchonereella bongraini*  *Tubeworm*  *Platynereis australis*  *Eunoe anderssoni*  *Barrukia cristata*  *Harmothoe spinose*  *Harmothoe sp.*  *Trypanosyllis gigantean*  *Ophelina syringopyge*  *Scoloplos (Leodamas) marginatus*  *Aglaophamus trissophyllus*  *Lumbrineris magalhaensis*  *Flabelligera gourdoni*  *Ilyphagus wyvillei*  *Pherusa kerguelarum*  *Flabelligeridae*  *Terebellidae*  *Eunoe opalina*  *Maupasia coeca*  *Tomopteris carpenteri*  *Tomopteris planktonis*  *Tomopteris septentrionalis*  *Tomopteris* | Bristle worms  NA  NA  NA  NA  Catworms  NA  NA  NA  Scale worms  NA  NA  NA  Bamboo worms  NA  NA  NA  NA  NA  NA  NA  NA  NA  NA  NA  NA  NA  NA  NA  NA  NA  NA  NA  NA  NA  NA  NA  NA  NA  NA  NA  NA | Class  Genus  Genus  Family  Genus  Family  Family  Genus  Family  Family  Genus  Family  Family  Family  Family  Family  Species  Family  Genus  Species  Family  Species  Species  Species  Species  Genus  Species  Species  Species  Species  Species  Species  Species  Species  Family  Family  Species  Species  Species  Species  Species  Genus |
| Ostracoda | Members of the class Ostracoda | *Ostracoda*  *Gigantocypris muelleri*  *Cytheridae*  *Gigantocypris sp.*  *Conchoecinae*  *Conchoecia sp.*  *Boroecia antipoda*  *Alacia belgicae*  *Austrinoecia isocheira*  *Alacia hettacra*  *Conchoecissa imbricata*  *Metaconchoecia skogsbergi* | Ostracods  NA  NA  NA  NA  NA  NA  NA  NA  NA  NA  NA | Class  Species  Family  Genus  Subfamily  Genus  Species  Species  Species  Species  Species  Species |
| Mysida | Members of the class Mysida | *Mysida*  *Antarctomysis maxima*  *Antarctomysis sp.*  *Antarctomysis ohlinii*  *Mysidae*  *Boreomysis rostrata*  *Boreomysis sp.*  *Euchaetomera zurstrasseni*  *Mysidetes morbihanensis*  *Mysidopsis sp.*  *Pseudomma sp.*  *Dactylamblyops sp.*  *Mysidetes posthon*  *Pseudomma belgicae* | Mysids  NA  NA  NA  NA  NA  NA  NA  NA  NA  NA  NA  NA  NA | Order  Species  Genus  Species  Family  Species  Genus  Species  Species  Genus  Genus  Genus  Species  Species |
| Pteropoda | Members of the order Pteropoda | *Pteropoda*  *Limacina sp.*  *Clio sp.*  *Limacina helicina*  *Clio pyramidata* | Pteropods  Sea snail  Sea butterfly  Sea snail  Sea butterfly | Order  Genus  Genus  Species  Species |
| Amphipoda | Members of the order Amphipoda | *Amphipoda*  *Hyperiella sp.*  *Hyperiidea*  *Cyllopus lucasii*  *Cyllopus magellanicus*  *Themisto gaudichaudii*  *Gammaridea*  *Waldeckia obesa*  *Cyllopus sp.*  *Hyperiidae*  *Lysianassidae*  *Oradarea sp.*  *Abyssorchomene plebs*  *Orchomenella sp.*  *Vibilia antarctica*  *Eurythenes gryllus*  *Podocerus capillimanus*  *Gammarellidae*  *Gondogeneia antarctica*  *Gondogeneia sp.*  *Eusiridae*  *Phoxocephalidae*  *Stenothoidae*  *Ischyroceridae*  *Jassa sp.*  *Corophiidea*  *Cheirimedon femoratus*  *Hippomedon kergueleni*  *Orchomenella (Orchomenopsis) rotundifrons*  *Cyphocaris sp.*  *Vibilia sp.*  *Hyperoche sp.*  *Hyperoche medusarum*  *Hyperiella antarctica*  *Hyperia macrocephala*  *Ampelisca richardsoni*  *Gammaropsis sp.*  *Joubinella sp.*  *Lepidepecreum urometacarinatum*  *Liljeborgia georgiana*  *Metopoides sp.*  *Monoculodes sp.*  *Oediceroides lahillei*  *Oediceroides sp.*  *Pseudericthonius gaussi*  *Rhachotropic schellenbergi*  *Tiron antarcticus*  *Uristes gigas*  *Zygomaera pfefferi*  *Byblis securiger*  *Eurythenes obesus*  *Eusirus perdentatus*  *Eusirus antarcticus*  *Ampelisca sp.*  *Epimeriidae*  *Haploops sp.*  *Oedicerotidae*  *Paraceradocus sp.*  *Rhachotropis antarctica*  *Synopiidae*  *Primno macropa*  *Chosroes decorates*  *Eurythenes sp.*  *Eusirus sp.*  *Paramoera walkeri*  *Cheirimedon fougneri*  *Epimeriella macronyx*  *Epimeriella walker*  *Cheirimedon solidus*  *Waldeckia sp.*  *Caprellidae*  *Vibilia antarctica*  *Hyperiella dilatata*  *Phronima sedentaria*  *Scina sp.*  *Jassa falcate*  *Eophiliantidae*  *Shackletonia sp.*  *Hyale sp.*  *Prostebbingia brevicornis*  *Nauticaris marionis*  *Polycheria kergueleni*  *Paramoera fissicauda*  *Cicadosa cicadoides*  *Hyperoche luetkenides*  *Cyphocaris richardi*  *Hyperia gaudichaudii*  *Hyperia spinigera*  *Vibilia propinqua*  *Parawaldeckia kidder*  *Podocerus capillimanus*  *Oedicerotidae*  *Megalanceola sp.*  *Gondogeneia ushuaiae*  *Ischyroceridae*  *Parandania boecki*  *Eusirus propeperdentatus*  *Leucothoe sp.*  *Seba antarctica*  *Iphimediidae*  *Sebidae*  *Orchomene sp.*  *Eusirus microps*  *Abyssorchomene sp.*  *Liljeborgia sp.*  *Hippomedon sp.*  *Lepidepecreum urometacarinatum*  *Tryphosella macropareia*  *Tryphosella intermedia*  *Rhachotropis sp.*  *Platyscelus ovoides*  *Eupronoe minuta*  *Vibilia armata*  *Lestrigonus sp.*  *Vibilia stebbingi*  *Cyphocaris faurei*  *Gnathiphimedia mandibularis*  *Epimeria similis*  *Epimeria georgiana*  *Echiniphimedia hodgsoni*  *Iphimediella sp.*  *Pseudorchomene coatsi*  *Pseudorchomene plebs*  *Hyperiella macronyx*  *Abyssorchomene rossi*  *Eusirus tridentatus*  *Cyllopus lucasii*  *Debroyerella solidus*  *Pseudorchomene rossi* | Amphipods  NA  NA  NA  NA  NA  NA  NA  NA  NA  NA  NA  NA  NA  NA  NA  NA  NA  NA  NA  NA  NA  NA  NA  NA  NA  NA  NA  NA  NA  NA  NA  NA  NA  NA  NA  NA  NA  NA  NA  NA  NA  NA  NA  NA  NA  NA  NA  NA  NA  NA  NA  NA  NA  NA  NA  NA  NA  NA  NA  NA  NA  NA  NA  NA  NA  NA  NA  NA  NA  NA  NA  NA  NA  NA  NA  NA  NA  NA  NA  NA  NA  NA  NA  NA  NA  NA  NA  NA  NA  NA  NA  NA  NA  NA  NA  NA  NA  NA  NA  NA  NA  NA  NA  NA  NA  NA  NA  NA  NA  NA  NA  NA  NA  NA  NA  NA  NA  NA  NA  NA  NA  NA  NA  NA  NA  NA  NA  NA  NA | Order  Genus  Suborder  Species  Species  Species  Suborder  Species  Genus  Family  Family  Genus  Species  Genus  Species  Species  Species  Family  Species  Genus  Family  Family  Family  Family  Genus  Family  Species  Species  Species  Genus  Genus  Genus  Species  Species  Species  Species  Genus  Genus  Species  Species  Genus  Genus  Species  Genus  Species  Species  Species  Species  Species  Species  Species  Species  Species  Genus  Family  Genus  Family  Genus  Species  Family  Species  Species  Genus  Genus  Species  Species  Species  Species  Species  Genus  Family  Species  Species  Species  Genus  Species  Family  Genus  Genus  Species  Species  Species  Species  Species  Species  Species  Species  Species  Species  Species  Species  Family  Genus  Species  Family  Species  Species  Genus  Species  Family  Family  Genus  Species  Genus  Genus  Genus  Species  Species  Species  Genus  Species  Species  Species  Genus  Species  Species  Species  Species  Species  Species  Genus  Species  Species  Species  Species  Species  Species  Species  Species  Species |
| Chaetognatha | Members of the Phylum Chaetognatha | *Chaetognatha*  *Pseudosagitta gazellae*  *Eukrohnia hamata*  *Sagitta sp.*  *Solidosagitta marri* | Arrow worms  NA  NA  NA  NA | Phylum  Species  Species  Genus  Species |
| Coelenterata | Species/Groups that can be classified as Coelenterata | *Siphonophorae*  *Hydrozoa*  *Cnidaria*  *Nematocysts*  *Hydroidolina*  *Scyphozoa*  *Ctenophora*  *Corymorpha sp.*  *Campanulariidae*  *Thaliacea*  *Halecium sp.*  *Pyrosoma sp.*  *Ulmaridae*  *Oswaldella antarctica*  *Ectopleura crocea* | Siphonophores  NA  NA  NA  NA  NA  Comb Jellies  NA  NA  NA  NA  NA  Jellyfish  NA  NA | Order  Class  Phylum  NA  Subclass  Class  Phylum  Genus  Family  Class  Genus  Genus  Family  Species  Species |
| Copepods | Members of the subclass Copepoda | *Copepoda*  *Calanoides acutus*  *Metridia gerlachei*  *Oncaea sp.*  *Oithona sp.*  *Harpacticoida*  *Calanidae*  *Calanoida*  *Metridia sp.*  *Calanus propinquus*  *Rhincalanus gigas*  *Microcalanus sp.*  *Clausocalanus laticeps*  *Gaetanus sp.*  *Paraeuchaeta sp.*  *Calanus simillimus*  *Drepanopus sp.*  *Euchaetidae*  *Ctenocalanus sp.*  *Candacia sp.*  *Drepanopus forcipatus*  *Paraeuchaeta antarctica*  *Haloptilus oxycephalus*  *Heterorhabdus sp.*  *Pleuromamma robusta*  *Scolecithricella sp.*  *Serolidae*  *Euchaeta sp.*  *Pleuromamma sp.*  *Aetideus sp.*  *Gaetanus tenuispinus*  *Haloptilus sp.*  *Scaphocalanus sp.*  *Onchocalanus sp.*  *Calanus sp.*  *Rhincalanus sp.*  *Clausocalanus sp.*  *Metrida lucens*  *Pleuromamma abdominalis*  *Pleuromamma sp.*  *Calanus australis*  *Lucicutia sp.*  *Corycaeus sp.*  *Oithona similis*  *Scaphocalanus vervoorti*  *Aetideus armatus*  *Undinella sp.*  *Stephos longipes*  *Ctenocalanus vanus*  *Cyclopoida*  *Drepanopus pectinatus*  *Sphyrion lumpi*  *Heterorhabdus austrinus*  *Lophoura sp.*  *Siphonostomatoida*  *Candacia maxima*  *Aetideopsis sp.*  *Neocalanus tonsus*  *Microcalanus pygmaeus*  *Euchirella rostromagna*  *Haloptilus ocellatus*  *Metridia curticauda*  *Triconia antarctica*  *Scaphocalanus farrani*  *Scolecithricella cenotelis*  *Spinocalanus abyssalis*  *Paralabidocera antarctica*  *Aetideopsis antarctica*  *Gaetanus kruppii*  *Paraeuchaeta similis*  *Oithona frigida*  *Oncaea curvata*  *Paraeuchaeta erebi*  *Gaetanus intermedius*  *Pseudochirella hirsuta*  *Pseudochirella mawsoni*  *Paraeuchaeta rasa*  *Paraheterorhabdus farrani*  *Lucicutia macrocera*  *Lucicutia gaussae*  *Lucicutia wolfendeni*  *Cephalophanes frigidus*  *Racovitzanus antarcticus*  *Scaphocalanus antarcticus*  *Scaphocalanus parantarcticus*  *Scolecithricella minor*  *Mimocalanus cultrifer*  *Spinocalanus abyssalis pygmaeus*  *Spinocalanus magnus* | Copepods  NA  NA  NA  NA  NA  NA  NA  NA  NA  NA  NA  NA  NA  NA  NA  NA  NA  NA  NA  NA  NA  NA  NA  NA  NA  NA  NA  NA  NA  NA  NA  NA  NA  NA  NA  NA  NA  NA  NA  NA  NA  NA  NA  NA  NA  NA  NA  NA  NA  NA  NA  NA  NA  NA  NA  NA  NA  NA  NA  NA  NA  NA  NA  NA  NA  NA  NA  NA  NA  NA  NA  NA  NA  NA  NA  NA  NA  NA  NA  NA  NA  NA  NA  NA  NA  NA  NA  NA | Subclass  Species  Species  Genus  Genus  Order  Family  Order  Genus  Species  Species  Genus  Species  Genus  Genus  Species  Genus  Family  Genus  Genus  Species  Species  Species  Genus  Species  Genus  Family  Genus  Genus  Genus  Species  Genus  Genus  Genus  Genus  Genus  Genus  Species  Species  Genus  Species  Genus  Genus  Species  Species  Species  Genus  Species  Species  Order  Species  Species  Species  Genus  Order  Species  Genus  Species  Species  Species  Species  Species  Species  Species  Species  Species  Species  Species  Species  Species  Species  Species  Species  Species  Species  Species  Species  Species  Species  Species  Species  Species  Species  Species  Species  Species  Species  Species  Species |
| Salps | Members of the order Salpidae | *Salpida*  *Salpa thompsoni*  *Salpidae*  *Salpa sp.* | Salps  NA  NA  NA | Order  Species  Order  Genus |
| Other krill | Members of the order Euphausiacea (excluding Antarctic krill) | *Thysanoessa macrura*  *Euphausia frigida*  *Euphausia triacantha*  *Euphausia lucens*  *Nematoscelis megalops*  *Thysanoessa gregaria*  *Thysanoessa sp.*  *Euphausia spinifera*  *Euphausia similis*  *Thysanoessa vivina*  *Stylocheiron sp.*  *Nyctiphanes australis*  *Nyctiphanes capensis*  *Stylocheiron* | NA  NA  NA  NA  NA  NA  NA  NA  NA  NA  NA  NA  NA  NA | Species  Species  Species  Species  Species  Species  Genus  Species  Species  Species  Genus  Species  Species  Genus |
| Antarctic krill | *Euphausia superba* | *Euphausia superba* | Antarctic krill | Species |
| Myctophids | Members of the family Myctophidae | *Myctophidae*  *Gymnoscopelus nicholsi*  *Electrona antarctica*  *Gymnoscopelus braueri*  *Gymnoscopelus fraseri*  *Electrona carlsbergi*  *Krefftichthys anderssoni*  *Protomyctophum bolini*  *Protomyctophum choriodon*  *Nannobrachium achirus*  *Protomyctophum normani*  *Gymnoscopelus opisthopterus*  *Protomyctophum sp.*  *Protomyctophum tenisoni*  *Lampichthys procerus*  *Gymnoscopelus microlampas*  *Gymnoscopelus bolini*  *Gymnoscopelus sp.*  *Gymnoscopelus hintonoides*  *Electrona subaspera*  *Diaphus sp.*  *Electrona sp.*  *Gymnoscopelus piabilis*  *Metelectrona ventralis*  *Protomyctophum andriashevi*  *Protomyctophum luciferum*  *Lampanyctus speculigera*  *Lampanyctus intricarius*  *Lampanyctus sp.*  *Nannobrachium achirus*  *Lampanyctodes hectoris*  *Symbolophorus sp.*  *Hintonia sp.*  *Electrona paucirastra*  *Metelectrona herwigi*  *Diaphus taaningi*  *Diaphus hudsoni*  *Diaphus effulgens*  *Diaphus luetkeni*  *Diaphus meadi*  *Benthosema suborbitale*  *Ceratoscopelus warmingii*  *Symbolophorus boops*  *Symboloporus barnardi*  *Hygophum hanseni*  *Hygophum hygomii*  *Lampanyctus alatus*  *Lampanyctus australis*  *Lampanyctus pusillus*  *Lobianchia dofleini*  *Lampadena pontifex*  *Diogenichthys atlanticus*  *Scopelopsis multipunctatus*  *Notoscopelus resplendens*  *Lampichthys procerus* | Lanternfish  Nichol’s lanternfish  Antarctic lanternfish  Brauer’s lanternfish  Fraser’s lanternfish  Electron subantarctic lanternfish  Rhombic lanternfish  Bolin’s lanternfish  Gaptooth lanternfish  NA  Norman’s lanternfish  NA  NA  Tenison’s lanternfish  Blackhead lanternfish  Minispotted lanternfish  Grand lanternfish  NA  False-midas lanternfish  Rough lanternfish  NA  NA  Southern blacktip lanternfish  Flaccid lanternfish  Andriashev’s lanternfish  Damsel lanternfish  NA  Diamondcheek lanternfish  NA  NA  Hector’s lanternfish  NA  NA  Belted lanternfish  Herwig lanternfish  Slopewater lanternfish  Hudson’s lanternfish  Headlight fish  Luetken’s lanternfish  Problematic lanternfish  Smallfin lanternfish  Warming’s lanternfish  Bogue lanternfish  Barnards lanterfish  Hansen’s lanternfish  Bermuda lanternfish  Winged lanternfish  Southern lanternfish  Pygmy lanternfish  Dofleini’s lanternfish  Jordans lanternfish  Longfin lanternfish  Multispotted lanternfish  Patchwork lampfish  Blackhead lanternfish | Family  Species  Species  Species  Species  Species  Species  Species  Species  Species  Species  Species  Genus  Species  Species  Species  Species  Genus  Species  Species  Genus  Genus  Species  Species  Species  Species  Species  Species  Genus  Species  Species  Genus  Genus  Species  Species  Species  Species  Species  Species  Species  Species  Species  Species  Species  Species  Species  Species  Species  Species  Species  Species  Species  Species  Species  Species |
| Antarctic silverfish | *Pleuragramma antarcticum* | *Pleuragramma antarcticum* | Antarctic silverfish | Species |
| Squid & Octopus | Members of the class *Cephalopoda* | *Sepiolidae*  *Psychroteuthis glacialis*  *Teuthida*  *Galiteuthis armata*  *Moroteuthis knipovitchi*  *Kondakovia longimana*  *Chiroteuthis sp.*  *Octopoda*  *Gonatus antarcticus*  *Chiroteuthis veranii*  *Brachioteuthis sp.*  *Adelieledone polymorpha*  *Pareledone sp.*  *Pareledone turqueti*  *Galiteuthis glacialis*  *Octopodidae*  *Loligo sp.*  *Ancistrocheirus lesueurii*  *Octopoteuthis rugosa*  *Taningia danae*  *Moroteuthis ingens*  *Moroteuthis robsoni*  *Onychoteuthis banksii*  *Pholidoteuthis massyae*  *Batoteuthis skolops*  *Histioteuthis sp.*  *Histioteuthis eltaninae*  *Histioteuthis atlantica*  *Psychroteuthis sp.*  *Architeuthis sp.*  *Illex sp.*  *Martialia hyadesi*  *Chiroteuthis imperator*  *Lepidoteuthis grimaldii*  *Mastigoteuthis sp.*  *Discoteuthis sp.*  *Egea inermis*  *Galiteuthis sp.*  *Helicocranchia sp.*  *Megalocranchia sp.*  *Mesonychoteuthis hamiltoni*  *Taonius pavo*  *Taonius sp.*  *Teuthowenia sp.*  *Haliphron atlanticus*  *Vampyroteuthis infernalis*  *Illex argentinus*  *Loligo gahi*  *Loligo sanpaulensis*  *Brachioteuthis picta*  *Nototeuthis dimegacotyle*  *Mastigoteuthis psychrophila*  *Onychoteuthidae*  *Histioteuthis macrohista*  *Decapodiformes*  *Mastigoteuthis psychrophila*  *Todarodes sagittatus*  *Mesonychoteuthis sp.*  *Enoploteuthidae*  *Octopoteuthis sp.*  *Histioteuthis meleagroteuthis*  *Histioteuthis bonnellii*  *Histioteuthis dofleini*  *Gonatus sp.*  *Chiroteuthidae*  *Architeuthidae*  *Cephalophoxoides sp.*  *Slosarczykovia circumantarctica*  *Todarodes sp.*  *Histioteuthis Miranda*  *Ommastrephes bartramii*  *Thaumeledone gunteri*  *Batoteuthis sp.*  *Liocranchia sp.*  *Todarodes filippovae*  *Brachioteuthis riisei*  *Enteroctopus magnificus*  *Oegopsida*  *Megalocranchia maxima*  *Histioteuthis bruuni*  *Cycloteuthis sirventi*  *Discoteuthis discus*  *Batoteuthidae*  *Lycoteuthis sp.*  *Moroteuthis sp.*  *Teuthowenia pellucida*  *Brachioteuthidae*  *Brachioteuthis linkovskyi*  *Todarodes angolensis*  *Ommastrephidae*  *Benthoctopus thielei*  *Onychoteuthis sp.*  *Stauroteuthis gilchristi*  *Opisthoteuthis sp.*  *Cirrata sp.*  *Octopodiformes*  *Octopus sp.*  *Architeuthis dux*  *Cycloteuthis akimushkini*  *Mastigoteuthidae*  *Cycloteuthis sp.*  *Pholidoteuthis sp.*  *Chiroteuthis mega*  *Taonius belone*  *Cranchiidae*  *Histioteuthidae*  *Alluroteuthis antarcticus*  *Lycoteuthis lorigera*  *Stoloteuthis sp.*  *Alluroteuthis sp.*  *Lampadioteuthis sp.*  *Moroteuthis hyadesi*  *Enoploteuthis sp.*  *Bathyteuthis abyssicola*  *Onykia ingens*  *Filippovia knipovitvhi*  *Lycoteuthis*  *Onykia robsoni*  *Stigmatoteuthis dofleini*  *Muusoctopus thielei* | Bobtail squid  Glacial squid  Squid  Armed cranch squid  Smooth hooked squid  Giant warty squid  NA  Octopus  NA  Long-armed squid  NA  NA  NA  Turquet’s octopus  Glass squid  NA  NA  Sharpear enope squid  NA  Dana octopus squid  Greater hooked squid  Rugose hooked squid  Common clubhook squid  NA  Bush-club squid  NA  NA  NA  NA  NA  NA  Sevenstar flying squid  NA  Grimaldi scaled squid  NA  NA  Glass squid  NA  NA  NA  Colossal squid  Glass squid  NA  NA  Seven-arm octopus  Vampire squid  Argentine shortfin squid  NA  NA  NA  NA  Whip-lash squid  NA  NA  NA  Whip-lash squid  NA  NA  NA  NA  NA  Umbrella squid  NA  NA  NA  NA  NA  NA  NA  NA  Neon flying squid  NA  NA  NA  NA  Common arm squid  Southern giant octopus  NA  NA  NA  NA  NA  NA  NA  NA  Googly-eyed glass squid  NA  NA  NA  NA  NA  NA  NA  NA  NA  NA  NA  Giant squid  NA  Whip-lash squid  NA  NA  NA  Glass squid  NA  NA  Antarctic neosquid  NA  NA  NA  NA  NA  NA  Deepsea squid  Greater hooked squid  Smooth hooked squid  NA  Rugose hooked squid  NA  NA | Family  Species  Order  Species  Species  Species  Genus  Order  Species  Species  Genus  Species  Genus  Species  Species  Family  Genus  Species  Species  Species  Species  Species  Species  Species  Species  Genus  Species  Species  Genus  Genus  Genus  Species  Species  Species  Genus  Genus  Species  Genus  Genus  Genus  Species  Species  Genus  Genus  Species  Species  Species  Species  Species  Species  Species  Species  Family  Species  Superorder  Species  Species  Genus  Family  Genus  Species  Species  Species  Genus  Family  Family  Genus  Species  Genus  Species  Species  Species  Genus  Genus  Species  Species  Species  Order  Species  Species  Species  Species  Family  Genus  Genus  Species  Family  Species  Species  Family  Species  Genus  Species  Genus  Genus  Superorder  Genus  Species  Species  Family  Genus  Genus  Species  Species  Family  Family  Species  Species  Genus  Genus  Genus  Species  Genus  Species  Species  Species  Genus  Species  Species  Species |
| Bathypelagic fish | Fish found in the bathypelagic zone | *Phosichthys argenteus*  *Scopelosaurus ahlstromi*  *Scopelosaurus hamiltoni*  *Notolepis sp.*  *Notolepis coatsi*  *Paralepididae*  *Magnisudi prionosa*  *Arctozenus risso*  *Magnisudis sp.*  *Bathylagus sp.*  *Bathylagus antarcticus*  *Nansenia antarctica*  *Microstomatidae*  *Bathylagus tenuis*  *Bathylagus sp.*  *Melamphaes sp.*  *Sio nordenskjoldii*  *Melamphaidae*  *Poromitra crassiceps*  *Muraenolepis microps*  *Muraenolepis sp.*  *Muraenolepis marmoratus*  *Muraenolepididae*  *Muraenolepis orangiensis*  *Macrouridae*  *Macrourus holotrachys*  *Macrourus sp.*  *Ventrifossa nasuta*  *Lepidorhynchus denticulatus*  *Moridae*  *Antimora rostrata*  *Anotopterus pharao*  *Pseudoscopelus sp.*  *Anotopterus vorax*  *Pseudoicichthys australis*  *Melanostigma gelatinosum*  *Melanonus gracilis*  *Maurolicus muelleri*  *Melamphaes sp.*  *Cynomacrurus piriei*  *Stomias gracilis* | Silver lightfish  Waryfish  Waryfish  NA  Antarctic jonasfish  Barracudina  NA  Spotted barracudina  NA  NA  Antarctic deepsea smelt  NA  Marine smelts  NA  NA  NA  Nordenskjold’s bigscale  Ridgeheads  Crested bigscale  Smalleye moray cod  NA  Marbled moray cod  Eel-cods  Patagonian moray cod  Grenadiers  Bigeye grenadier  NA  Conesnout grenadier  Thorntooth grenadier  NA  Blue antimora  Daggertooth  NA  South Ocean Daggertooth  Southern driftfish  Limp eelpout  Pelagic cod  Silvery lightfish  NA  Dogtooth grenadier  NA | Species  Species  Species  Genus  Species  Family  Species  Species  Genus  Genus  Species  Species  Family  Species  Genus  Genus  Species  Family  Species  Species  Genus  Species  Family  Species  Family  Species  Genus  Species  Species  Family  Species  Species  Genus  Species  Species  Species  Species  Species  Genus  Species  Species |
| Cod icefish | Members of the family Nototheniidae | *Notothenioidei*  *Notothenia neglecta*  *Nototheniidae*  *Trematomus hansoni*  *Trematomus scotti*  *Trematomus sp.*  *Lepidonotothen nudifrons*  *Gobionotothen gibberfrons*  *Nototheniops nybelini*  *Lepidonotothen squamifrons*  *Lepidonotothen larseni*  *Notothenia coriiceps*  *Trematomus bernacchii*  *Trematomus eulepidotus*  *Trematomus newnesi*  *Notothenia rossii*  *Patagonotothen guntheri*  *Gobionotothen marionensis*  *Paranotothenia magellanica*  *Patagonotothen brevicauda*  *Gobionotothen angustifrons*  *Notothenia sp.*  *Pagothenia borchgrevinki*  *Trematomus loennbergii*  *Aethotaxis mitopteryx*  *Trematomus lepidorhinus*  *Trematomus pennellii*  *Gobionotothen acuta*  *Lepidonotothen mizops*  *Notothenia cyanobrancha*  *Lepidonotothen sp.* | NA  Yellowbelly rockcod  Cod icefishes  Striped rockcod  Crowned rockcod  NA  Yellowfin notie  NA  NA  Grey rockcod  Painted notie  Black rockcod  Emerald rockcod  Blunt scalyhead  Dusky rockcod  Marbled rockcod  Yellowfin notothen  Lobe-lip notothen  Magellanic rockcod  Patagonian rockcod  Narrowhead rockcod  NA  Bald notothen  Scaly rockcod  Longfin icedevil  Slender scalyhead  Sharp-Spined notothenia  Triangular rockcod  Toad notie  Blue rockcod  NA | Suborder  Species  Family  Species  Species  Genus  Species  Species  Species  Species  Species  Species  Species  Species  Species  Species  Species  Species  Species  Species  Species  Genus  Species  Species  Species  Species  Species  Species  Species  Species  Genus |
| Other Demersal Fish | Other species of fish found in the shelf region (i.e. demersal species) | *Channichthyidae*  *Chionodraco sp.*  *Pagetopsis macropterus*  *Pagetopsis sp.*  *Pagetopsis maculatus*  *Chaenodraco wilsoni*  *Neopagetopsis ionah*  *Channichthys rhinoceratus*  *Chaenocephalus aceratus*  *Chionodraco rhinoceratus*  *Chionodraco rastrospinosus*  *Cryodraco antarcticus*  *Pseudochaenichthys georgianus*  *Chionodraco hamatus*  *Chionodraco myersi*  *Dacodraco hunter*  *Parachaenichthys charcoti*  *Parachaenichthys georgianus*  *Melanostomiidae*  *Stomiidae*  *Bathydraconidae*  *Gymnodraco acuticeps*  *Cygnodraco mawsoni*  *Gerlachea australis*  *Pogonophryne scotti*  *Atedidraco sp.*  *Artedidraco orianae*  *Pogonophryne permitini*  *Artedidraconidae*  *Harpagifer antarcticus*  *Harpagifer georgianus*  *Harpagifer sp.*  *Harpagifer spinosus*  *Harpagifer kerguelensis*  *Harpagifer bispinis*  *Geotria australis*  *Petromyzontidae*  *Paradiplospinus gracilis*  *Gempylidae*  *Paradiplospinus sp.*  *Zoarcidae*  *Bathyraja sp.*  *Amblyraja georgiana*  *Mancopsetta maculata*  *Muraenolepis microps*  *Macrourus whitsoni*  *Chaunax sp.*  *Zanclorhynchus spinifer*  *Argentina elongate*  *Guttigadus kongi*  *Psilodraco georgianus*  *Bathydraco marri*  *Borostomias antarcticus*  *Vomeridens infuscipinnis*  *Artedidraco mirus*  *Artedidraco sp.*  *Etmopterus cf. granulosus*  *Echiodon cryomargarites*  *Coryphaenoides subserrulatus* | Crocodile icefish  NA  NA  NA  NA  Spiny icefish  Jonah’s icefish  Unicorn icefish  Blackfin icefish  NA  Ocellated icefish  Long-fingered icefish  South Georgia icefish  NA  Myer’s icefish  NA  NA  NA  NA  NA  Antarctic dragonfishes  Ploughfish  Mawson’s dragonfish  NA  Saddleback plunderfish  NA  NA  Finespotted plunderfish  Barbeled plunderfishes  Antarctic spiny plunderfish  South Georgia spiny plunderfish  NA  Deep-water spiny plunderfish  Kerguelen spiny plunderfish  Magellan plunderfish  Pouched lamprey  Lamprey  Slender escolar  Snake mackerals  NA  Eelpouts  NA  Antarctic starry skate  Antarctic armless flounder  Smalleye moray cod  Whitson’s grenadier  NA  Antarctic horsefish  NA  Austral cod  NA  Deep-water dragon  Snaggletooth  NA  NA  NA  Southern lanternshark  Messmate  Longrayed whiptail | Family  Genus  Species  Genus  Species  Species  Species  Species  Species  Species  Species  Species  Species  Species  Species  Species  Species  Species  Family  Family  Family  Species  Species  Species  Species  Genus  Species  Species  Family  Species  Species  Genus  Species  Species  Species  Species  Order  Species  Family  Genus  Family  Genus  Species  Species  Species  Species  Genus  Species  Species  Species  Species  Species  Species  Species  Species  Genus  Species  Species  Species |
| Mackeral icefish | *Champsocephalus gunnari* | *Champsocephalus gunnari* | Mackeral icefish | Species |
| Antarctic toothfish | *Dissostichus mawsoni* | *Dissostichus mawsoni* | Antarctic toothfish | Species |
| Patagonian toothfish | *Dissostichus eleginoides* | *Dissostichus eleginoides* | Patagonian toothfish | Species |
| Other seabirds | Other species of flying seabirds | *Phalacrocorax atriceps*  *Phalacrocorax atriceps nivalis*  *Larus dominicanus*  *Chionis minor*  *Pelecanoides georgicus*  *Pelecanoides urinatrix exsul*  *Pelecanoides urinatrix*  *Pelecanoides sp.*  *Sterna paradisaea*  *Sterna vittata*  *Phalacrocorax atriceps bransfieldensis*  *Phalacrocorax atriceps georgianus*  *Fregetta tropica*  *Oceanites oceanicus*  *Phalacrocorax albiventer purpurascens*  *Daption capense*  *Fulmarus glacialoides*  *Macronectes giganteus*  *Pachyptila vittata*  *Pagodroma nivea*  *Pterodroma inexpectata*  *Thalassoica antarctica*  *Puffinus griseus*  *Procellaria aequinoctialis*  *Pachyptila turtur*  *Pachyptila crassirostris*  *Pachyptila desolata*  *Procellariidae*  *Halobaena caerulea*  *Pterodroma brevirostris*  *Pachyptila belcheri*  *Pachyptila salvini*  *Macronectes halli*  *Procellaria aequinoctialis*  *Macronectes sp.*  *Puffinus tenuirostris*  *Pterodroma mollis*  *Pterodroma macroptera*  *Prions*  *Procellaria cinerea*  *Garrodia nereis* | Imperial shag  Imperial shag  Kelp gull  Black-faced sheathbill  South Georgia diving petrel  Common diving petrel  Common diving petrel  NA  Arctic tern  Antarctic tern  Antarctic shag  South Georgia shag  Black-bellied storm petrel  Wilson’s storm petrel  Imperial shag  Cape petrel  Southern fulmar  Southern giant petrel  Broad-billed prion  Snow petrel  Mottled petrel  Antarctic petrel  Sooty shearwater  White-chinned petrel  Fairy prion  Fulmar prion  Antarctic prion  NA  Blue petrel  Kerguelen petrel  Slender-billed prion  Salvin’s prion  Northern giant petrel  White-chinned petrel  NA  Short-tailed shearwater  Soft-plumaged petrel  Great-winged petrel  NA  Grey petrel  Grey-backed storm petrel | Species  Species  Species  Species  Species  Species  Species  Genus  Species  Species  Species  Species  Species  Species  Species  Species  Species  Species  Species  Species  Species  Species  Species  Species  Species  Species  Species  Family  Species  Species  Species  Species  Species  Species  Genus  Species  Species  Species  Tribe  Species  Species |
| Albatross | Members of the family *Diomedeidae* | *Diomedea exulans*  *Thalassarche chrysostoma*  *Thalassarche melanophris*  *Phoebetria palpebrata*  *Thalassarche chlororhynchos*  *Phoebetria fusca*  *Diomedeidae* | Wandering albatross  Grey-headed albatross  Black-browed albatross  Light-mantled albatross  Atlantic yellow-nosed albatross  Sooty albatross  Albatross | Species  Species  Species  Species  Species  Species  Family |
| Skuas | Members of the family Stercorariidae | *Catharacta lonnbergi*  *Catharacta Antarctica*  *Catharacta maccormicki*  *Stercorarius sp.*  *Stercorarius maccormicki* | Brown skua  Brown skua  South Polar skua  NA  South Polar skua | Species  Species  Species  Genus  Species |
| Emperor penguin | *Aptenodytes forsteri* | *Aptenodytes forsteri* | Emperor penguin | Species |
| Adelie penguin | *Pygoscelis adeliae* | *Pygoscelis adeliae* | Adelie penguin | Species |
| Chinstrap penguin | *Pygoscelis antarcticus* | *Pygoscelis antarcticus* | Chinstrap penguin | Species |
| Gentoo penguin | *Pygoscelis papua* | *Pygoscelis papua* | Gentoo penguin | Species |
| Macaroni penguin | *Eudyptes chrysolophus* | *Eudyptes chrysolophus* | Macaroni penguin | Species |
| King penguin | *Aptenodytes patagonicus* | *Aptenodytes patagonicus* | King penguin | Species |
| Rockhopper penguin | Rockhopper penguin species | *Eudyptes chrysocome*  *Eudyptes chrysocome filholi* | Southern rockhopper penguin  Eastern rockhopper penguin | Species  Species |
| Royal penguin | *Eudyptes schlegeli* | *Eudyptes schlegeli* | Royal penguin | Species |
| Antarctic fur seal | *Arctocephalus gazella* | *Arctocephalus gazella* | Antarctic fur seal | Species |
| Sub-Antarctic fur seal | *Arctocephalus tropicalis* | *Arctocephalus tropicalis* | Sub-Antarctic fur seal | Species |
| New Zealand sea lion | *Phocarctos hookeri* | *Phocarctos hookeri* | New Zealand sea lion | Species |
| Elephant seal | Members of the genus *Mirounga* | *Mirounga leonine*  *Mirounga sp.* | Southern Elephant seal  NA | Species  Genus |
| Weddell seal | *Leptonychotes weddellii* | *Leptonychotes weddellii* | Weddell seal | Species |
| Ross seal | *Ommatophoca rossii* | *Ommatophoca rossii* | Ross seal | Species |
| Crabeater seal | *Lobodon carcinophagus* | *Lobodon carcinophagus* | Crabeater seal | Species |
| Leopard seal | *Hydrurga leptonyx* | *Hydrurga leptonyx* | Leopard seal | Species |
| Dolphins & Ziphiids | Species classified as dolphins or ziphiids | *Globicephala melas*  *Hyperoodon planifrons*  *Cephalorhynchus commersonii* | Long-finned pilot whale  Southern bottlenose whale  Commerson’s dolphin | Species  Species  Species |
| Minke whales | Members of the genus *Balaenoptera* | *Balaenoptera acutorostrata*  *Balaenoptera bonaerensis* | Minke whale  Antarctic minke whale | Species  Species |
| Baleen whales | Baleen whale species | *Eubalaena australis*  *Balaenoptera musculus intermedia* | Humpback whale  Antarctic blue whale | Species  Species |
| Killer whales | *Orcinus orca* | *Orcinus orca* | Killer whale | Species |

**Table S3**: Functional group classification, definition and included groups from Table S2 used in the 15 functional-group simplified Southern Ocean food web dataset

| Functional group | Group definition | Species / groups included |
| --- | --- | --- |
| Phytoplankton | Phytoplankton | Phytoplankton |
| Benthos | All benthic groups | Sedentary benthos  Herbivorous benthos  Predatory benthos |
| Other zooplankton | All zooplankton groups excluding krill species and copepods | Eukaryotes  Tintinnia  Polychaeta  Ostracods  Mysida  Pteropods  Amphipods  Chaetognatha  Coelenterata  Salps |
| Other krill | Other krill | Other krill |
| Antarctic krill | Antarctic krill | Antarctic krill |
| Copepods | Copepods | Copepods |
| Demersal fish | All demersal fish groups | Cod icefish  Other demersal fish  Mackeral icefish  Antarctic toothfish  Patagonian toothfish |
| Mesopelagic fish | Myctophids  Antarctic silverfish | Myctophids  Antarctic silverfish |
| Cephalopods | Cephalopods | Cephalopods |
| Bathypelagic fish | Bathypelagic fish | Bathypelagic fish |
| Seabirds | All seabird groups | Other seabirds  Albatross  Skuas |
| Penguins | All penguin species/groups | Emperor penguin  Adelie penguin  Chinstrap penguin  Gentoo penguin  Macaroni penguin  King penguin  Rockhopper penguins  Royal penguin |
| Other seals | All seal penguins excluding leopard seals | Sub-Antarctic fur seal  Antarctic fur seal  New Zealand sea lion  Elephant seal  Weddell seal  Ross seal  Crabeater seal |
| Leopard seal | Leopard seal | Leopard seal |
| Whales | Minke whales | Minke whales |

**
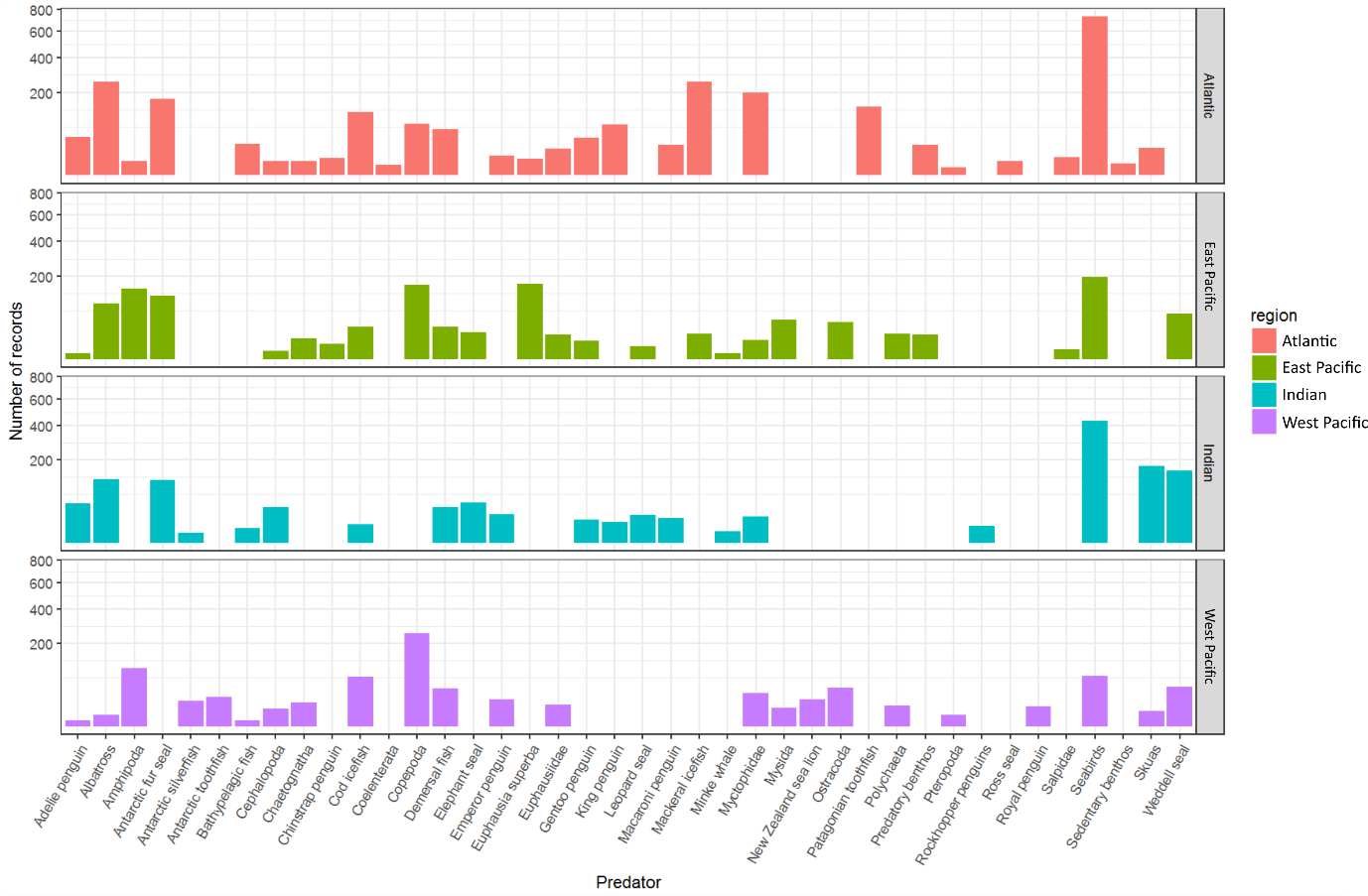
**

**Figure S1:** Number of records per individual predator species sampled in each of the four sectors of the Southern Ocean present in the SCAR Southern Ocean Diet and Energetics Database (SCAR 2018).

**
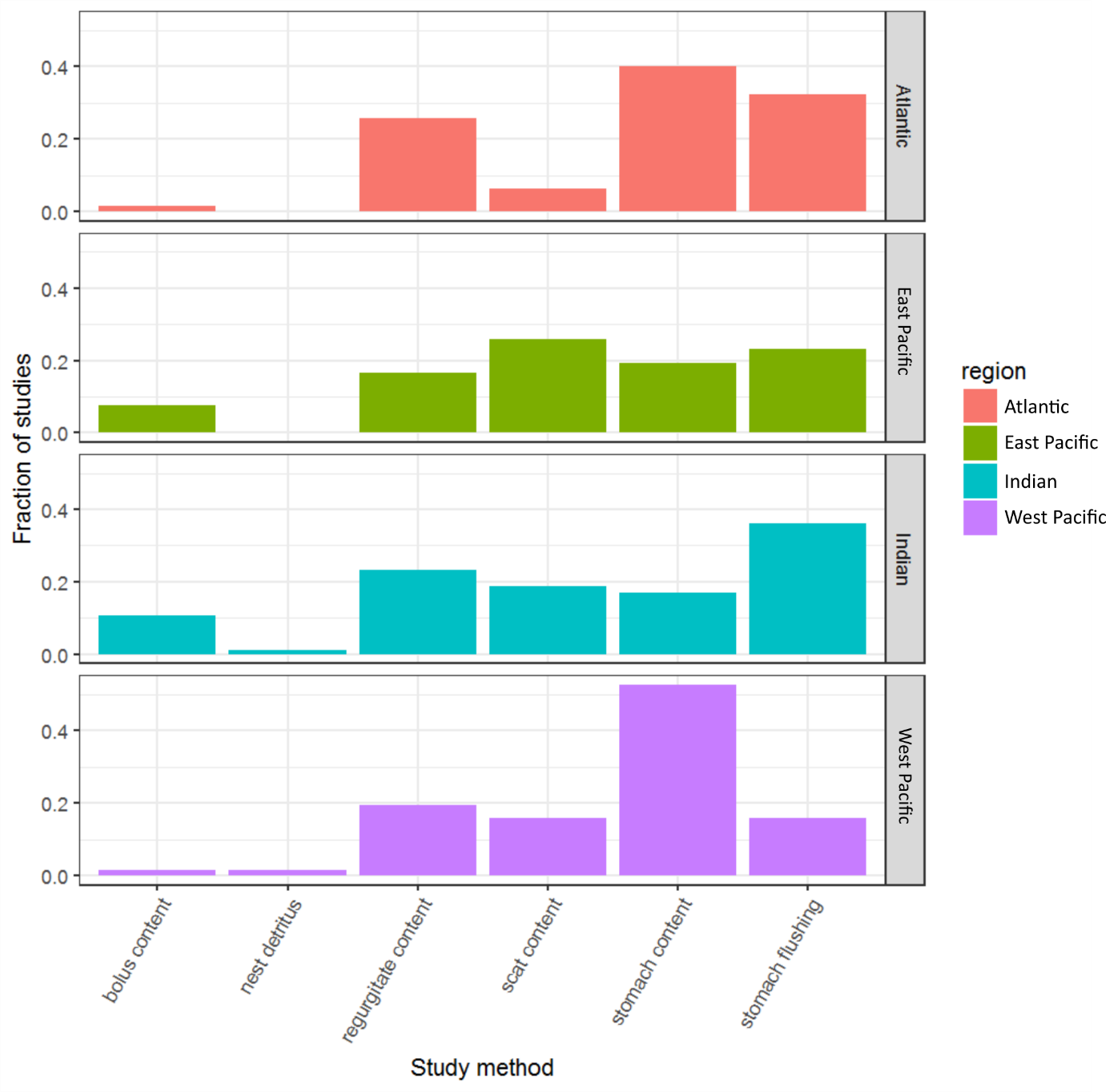
**

**Figure S2:** Fraction of total studies used in region-specific food web analyses for each study method within the SCAR Diet and Energetics Database by region.

**Species Accumulation Curves**

Using the classic method outlined by Gotelli and Colwell (2001) which finds the mean species accumulation curve and its standard deviation from random permutations of the data (or subsampling without replacement), we generated species accumulation curves for each sector-specific dataset (Fig. S3). For each sector, an asymptote was reached indicating that given the available data sources for each sector, each prey group received adequate coverage.

**
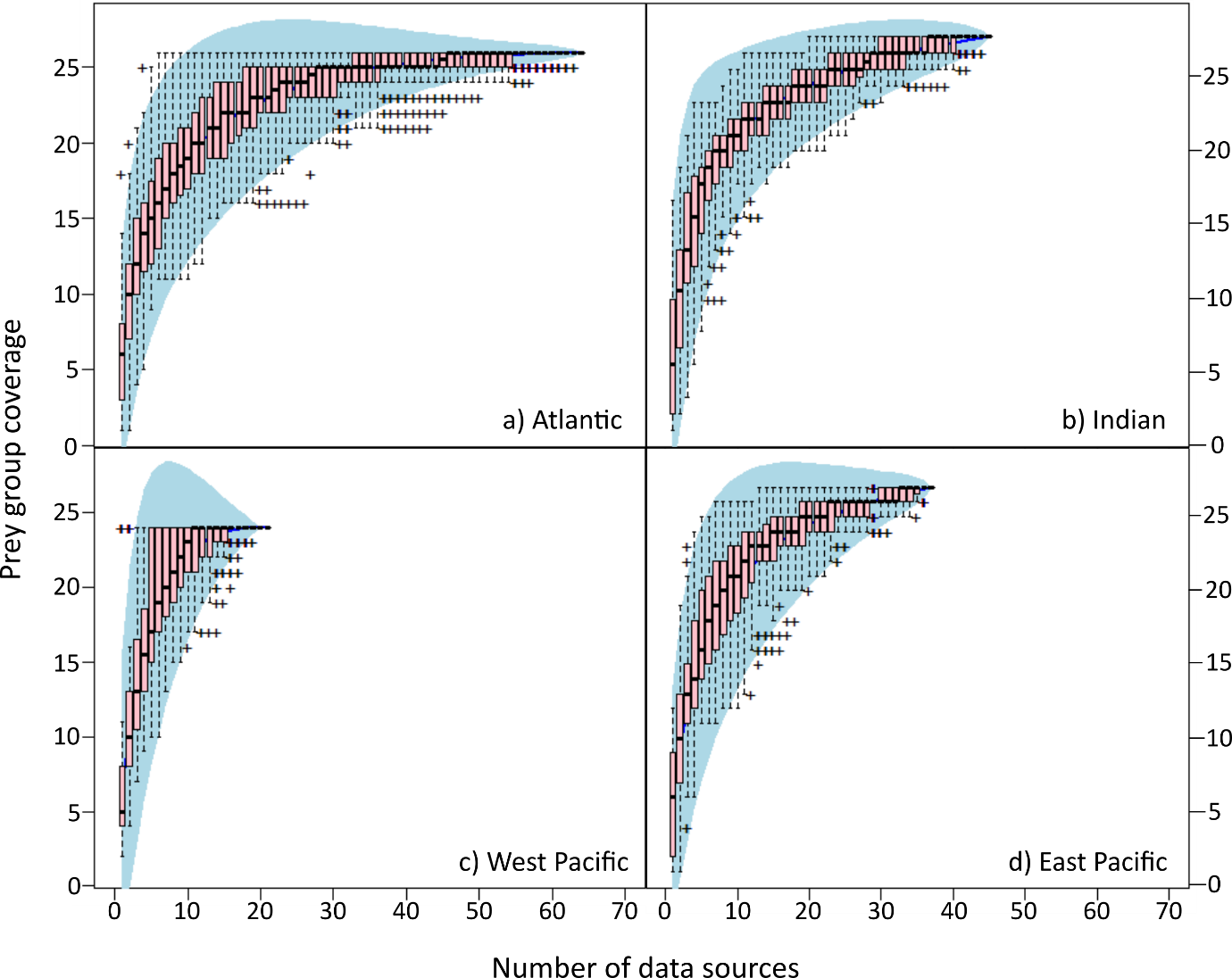
**

**Figure S3:** Species accumulation curves for each sector of the Southern Ocean indicating the number of individual data sources per sector by prey groups sampled. The plot indicates the lower (Q1) and upper (Q3) quartile for each random permutation, the mean number of prey groups sampled is shown by the thick black line and outliers are represented by the plus (+) symbol. The light blue shaded areas indicate 95% confidence intervals.

**References**

Gotelli, N. J., and R. K. Colwell. 2001. Quantifying biodiversity: procedures and pitfalls in the measurement and comparison of species richness. Ecology letters **4**:379-391.

Scientific Committee on Antarctic Research (SCAR). 2018. Southern ocean Diet and Energetics Database. doi: 10.26179/5b6cd40bb6935
